# Supplementary material for: Use of glucocorticoids megadoses in SARS-CoV-2 infection in a spanish registry: SEMI-COVID-19
Source: PLoS One. 2022 Jan 21;17(1):e0261711. doi: 10.1371/journal.pone.0261711 (PMC8782507; doi:10.1371/journal.pone.0261711)
Supplement: S1 File — (DOCX) [file pone.0261711.s001.docx]

Statistical results

Contents

[**1.**](#_heading=h.gjdgxs) Study variables **2**

[**2.**](#_heading=h.30j0zll) Descriptive **6**

[∙](#_heading=h.1fob9te) Numerical variables 6

[∙Categorical variables 7](#_heading=h.3znysh7)

[**3.**](#_heading=h.2et92p0) Bivariate tables 4 and 5 **20**

[∙](#_heading=h.tyjcwt) Categorical variables [20](#_heading=h.tyjcwt)

[∙](#_heading=h.3dy6vkm) Numerical variables [21](#_heading=h.3dy6vkm)

[**4.**](#_heading=h.1t3h5sf) Bivariate Exitus in income **23**

[∙](#_heading=h.4d34og8) Categorical variables [23](#_heading=h.4d34og8)

[∙](#_heading=h.2s8eyo1) Numerical variables [31](#_heading=h.2s8eyo1)

[**5.**](#_heading=h.17dp8vu) Bivariate Death during admission or re-admission **34**

[∙](#_heading=h.3rdcrjn) Categorical variables [34](#_heading=h.3rdcrjn)

[∙](#_heading=h.26in1rg) Numerical variables [42](#_heading=h.26in1rg)

[**6.**](#_heading=h.lnxbz9) Multivariate analysis **45**

[∙](#_heading=h.35nkun2) Exitus 45

[∙](#_heading=h.1ksv4uv) Died during admission 47

## Study variables

Contains data from CORTI_SEMI 2.dta

obs: 14.921

vars: 218 12 Mar 2021 10:42

------------------------------------------------------------------------------------------------------------------------

storage display value

variable name type format label variable label

------------------------------------------------------------------------------------------------------------------------

iniciale double %10.0g Referencia

fing float %td Fecha de ingreso

fecini float %td Fecha inicio de los síntomas

falt float %td Fecha de alta o exitus

motalt byte %33.0g dmotalt Motivo alta

estancia float %9.0g Días de estancia hospitalaria en el ingreso

sintomas_d float %9.0g Días de síntomas hasta ingreso

Exitus float %16.0g dNoSi Exitus en el ingreso

motex byte %26.0g dmotex Causa del éxitus

reingr byte %16.0g dNoSi Reingreso (no incluir al paciente como nuevo si ha reingresado)

creingr byte %16.0g dcreingr Causa del reingreso

freingr float %td Fecha Reingreso

reingr_dias float %9.0g Días desde al alta hasta reingreso

otcrein str199 %199s Especificar causa reingreso

farein float %td Fecha de alta o exitus tras reingreso

motaltr byte %33.0g dmotaltr Motivo alta tras reingreso

fallec byte %16.0g dNoSi Fallecimiento durante el ingreso o reingreso

motexr byte %26.0g dmotexr Causa del exitus tras reingreso

fallpos byte %16.0g dNoSi En caso de que el paciente no haya fallecido durante el ingreso o

reingreso, ¿S

fpcrul float %td Fecha última PCR SARS-CoV-2

rpcrul byte %16.0g drpcrul Resultado última PCR SARS-CoV-2

edad float %9.0g Edad

edad_d float %22.0g edad_d Edad por décadas

sexo byte %16.0g dsexo Sexo

rnegr byte %16.0g drnegr Raza

hta byte %16.0g dNoSi Hipertensión arterial

dm2 float %16.0g dNoSi Diabetes Mellitus

dmnlod byte %16.0g dNoSi Diabetes sin lesión de órgano diana

dmlod byte %16.0g dNoSi Diabetes con lesión en órgano diana

dislip byte %16.0g dNoSi Dislipemia

obesid byte %16.0g dNoSi Obesidad (IMC mayor o igual a 30 kg/m2)

peso float %9.0g Peso (kg)

tabaq byte %18.0g dtabaq Historia de tabaquismo

aboh byte %16.0g dNoSi Enolismo

fauri byte %16.0g dNoSi Fibrilación auricular

icc byte %16.0g dNoSi Insuficiencia cardiaca

infmioc byte %16.0g dNoSi Infarto de miocardio

angor byte %16.0g dNoSi Angina de pecho

cisq float %16.0g dNoSi Cardiopatía isquémica

epoc byte %16.0g dNoSi EPOC

fev1 float %9.0g FEV1 (%)

brocron byte %16.0g dNoSi Bronquitis crónica

asma byte %16.0g dNoSi Asma

saos byte %16.0g dNoSi Síndrome de apnea-hipopnea del sueño

acva float %16.0g dNoSi Ictus/AIT

ait byte %16.0g dNoSi Accidente isquémico transitorio o ictus isquémico sin secuelas

acvisq byte %16.0g dNoSi ACV isquémico o hemorrágico con secuelas

hemiplej byte %16.0g dNoSi Hemiplejia o paraplejia

demencia byte %16.0g dNoSi Demencia

neudeg byte %16.0g dNoSi Enfermedad neurológica degenerativa

hepato float %16.0g dNoSi Hepatopatía crónica

hepatlig byte %16.0g dNoSi Hepatopatía crónica leve

hepatopa byte %16.0g dNoSi Hepatopatía crónica moderada-severa

patrenal byte %16.0g dNoSi Insuficiencia renal crónica moderada-severa

hemod byte %23.0g dhemod Paciente habitualmente en diálisis

enfvaspe byte %16.0g dNoSi Enfermedad vascular periférica

enfulcer byte %16.0g dNoSi Úlcera gastroduodenal

cancer float %16.0g dNoSi Cáncer

neoplasi byte %16.0g dNoSi Neoplasia sólida sin metástasis

metsolid byte %16.0g dNoSi Neoplasia sólida con metástasis

leucemia byte %16.0g dNoSi Leucemia

linfoma byte %16.0g dNoSi Linfoma

conectiv byte %16.0g dNoSi Enfermedad del tejido conectivo

enrar byte %16.0g dNoSi Paciente con enfermedad rara/minoritaria

trans byte %16.0g dNoSi Trastorno de ansiedad

depres byte %16.0g dNoSi Depresión

sida byte %16.0g dNoSi SIDA (con criterios definitorios)

------------------------------------------------------------------------------------------------------------------------

storage display value

variable name type format label variable label

------------------------------------------------------------------------------------------------------------------------

infvih byte %16.0g dNoSi Infección VIH conocida previa (con o sin criterios de SIDA)

charlson byte %8.0g Índice de Charlson

chcored byte %8.0g Charlson corregido por edad

inmunodep float %16.0g dNoSi Inmunodepresores tto habitual

inmdep byte %22.0g dinmdep Tratamiento habitual con inmunosupresores (si está con varios, elegir

otros e in

rapam byte %16.0g dNoSi Rapamicina (Sirolimus)

tbiol byte %16.0g dNoSi Terapias biológicas de base (anticuerpos monoclonales)

corhab byte %16.0g dNoSi Tratamiento habitual con corticoides sistémicos

corinh byte %16.0g dNoSi Tratamiento habitual con corticoides inhalados

spo2 byte %8.0g Saturación de oxígeno pulsioximetría (%)

spo2_90 float %9.0g spo2_90 SatO2

fispo2 byte %25.0g dfispo2 FiO2 con la que se ha obtenido la SpO2

otfio2 byte %8.0g FiO2 real si se dispone de ella (%)

phin float %9.0g pH en sangre arterial al ingreso

pco2in float %9.0g PCO2 al ingreso (mmHg)

po2in float %9.0g PO2 al ingreso (mmHg)

pafi float %9.0g pO2/FiO2 al ingreso (mmHg) (PO2/FiO2(%) x 100)

oxsupl byte %25.0g doxsupl FiO2 con la que se ha obtenido la gasometría

otfio2s byte %8.0g FiO2 real si se dispone de ella (%)

spo2r byte %8.0g Saturación de oxígeno pulsioximetría (%)

spo2_90r float %9.0g spo2_90 SatO2 posterior

fispo2r byte %25.0g dfispo2r FiO2 con la que se ha obtenido la SpO2

otfio2r byte %8.0g FiO2 real si se dispone de ella (%)

phinr float %9.0g pH en sangre arterial

pco2inr float %9.0g PCO2 (mmHg)

po2inr float %9.0g PO2 (mmHg)

pafir float %9.0g pO2/FiO2 (mmHg) (PO2/FiO2(%) x 100)

oxsuplr byte %25.0g doxsuplr FiO2 con la que se ha obtenido la gasometría

otfio2sr byte %8.0g FiO2 real si se dispone de ella (%)

conden byte %16.0g dconden Condensación

inters byte %16.0g dinters Infiltrado intersticial/vidrio deslustrado

derpl byte %16.0g dderpl Derrame pleural

tctor byte %16.0g dNoSi ¿Realizada TC torácica?

condenr byte %16.0g dcondenr Condensación

intersr byte %16.0g dintersr Infiltrado intersticial/vidrio deslustrado

derplr byte %16.0g dderplr Derrame pleural

emprx byte %16.0g dNoSi ¿Empeoramiento radiológico?

condens float %16.0g dNoSi Condensación al ingreso

interst float %16.0g dNoSi Infiltrado intesticial al ingreso

derple float %16.0g dNoSi Derrame pleural al ingreso

condensr float %16.0g dNoSi Condensación en la evolución

interstr float %16.0g dNoSi Infiltrado intesticial en la evolución

derpler float %16.0g dNoSi Derrame pleural en la evolución

hb float %9.0g Hemoglobina (g/dL)

leuin long %12.0g Recuento de leucocitos (x10^6/L de sangre) al ingreso

neutin long %12.0g Recuento absoluto de neutrófilos (x10^6/L de sangre) al ingreso

linfin long %12.0g Recuento absoluto de linfocitos (x10^6/L de sangre) al ingreso

plaq long %12.0g Plaquetas (x 10^6/L)

pcrin float %9.0g Proteína C reactiva al ingreso (mg/L)

proca float %9.0g Procalcitonina (ng/mL)

ferrit float %9.0g Ferritina sérica (mcg/L)

dimerd long %12.0g Dímero D (ng/mL)

il6 float %9.0g Interleukina-6 (IL-6) (pg/mL)

ldh int %8.0g LDH (U/L)

lsldh int %8.0g Límite superior normalidad LDH en su centro (U/L)

creain float %9.0g Creatinina sérica al ingreso (mg/dL)

urea int %8.0g Urea (mg/dL)

hbr float %9.0g Hemoglobina (g/dL)

leuinr long %12.0g Recuento de leucocitos (x10^6/L de sangre)

neutinr long %12.0g Recuento absoluto de neutrófilos (x10^6/L de sangre)

linfinr long %12.0g Recuento absoluto de linfocitos (x10^6/L de sangre)

plaqr long %12.0g Plaquetas (x 10^6/L)

pcrinr float %9.0g Proteína C reactiva (mg/L)

procar float %9.0g Procalcitonina (ng/mL)

dimerdr long %12.0g Dímero D (ng/mL)

il6r float %9.0g Interleukina-6 (IL-6) (pg/mL)

ldhr int %8.0g LDH (U/L)

creainr float %9.0g Creatinina sérica (mg/dL)

urear int %8.0g Urea (mg/dL)

gotr int %8.0g GOT-AST(U/L)

gptr int %8.0g GPT-ALT (U/L)

glubasr int %8.0g Glucemia basal(mg/dL)

hb_12 float %9.0g hb_12 Hemoglobina (12 g/dL)

hbr_12 float %9.0g hb_12 Hemoglobina (12 g/dL)

leuin_10 float %10.0g leuin_10 Leucocitosis (10 x 10^6/L)

leuin_4 float %9.0g leuin_4 Leucocitos (4 x 10^6/L)

------------------------------------------------------------------------------------------------------------------------

storage display value

variable name type format label variable label

------------------------------------------------------------------------------------------------------------------------

leuinr_10 float %10.0g leuin_10 Leucocitosis (10 x 10^6/L)

leuinr_4 float %9.0g leuin_4 Leucocitos (4 x 10^6/L)

linfin_1300 float %9.0g linfin_1300

Linfocitos (1.3 x 10^6/L)

linfinr_1300 float %9.0g linfin_1300

Linfocitos (1.3 x 10^6/L)

plaq_180 float %9.0g plaq_180 Plaquetas (180 x 10^6/L)

plaqr_180 float %9.0g plaq_180 Plaquetas (180 x 10^6/L)

pcrin_50 float %17.0g pcrin_50 PCR (50 mg/L)

pcrinr_50 float %17.0g pcrin_50 PCR (50 mg/L)

proca_05 float %9.0g proca_05 Procalcitonina (0.5 ng/mL)

procar_05 float %9.0g proca_05 Procalcitonina (0.5 ng/mL)

ferrit_1000 float %9.0g ferrit_1000

Ferritina (1000 mcg/L)

ferrit_274 float %9.0g ferrit_274

Ferritina (274 mcg/L)

dimerd_250 float %9.0g dimerd_250

Dímero D (250 ng/mL)

dimerdr_250 float %9.0g dimerd_250

Dímero D (250 ng/mL)

il6_43 float %9.0g il6_43 IL6 (4.3 pg/mL)

il6r_43 float %9.0g il6_43 IL6 (4.3 pg/mL)

ldh_300 float %9.0g ldh_300 LDH (300 U/L)

ldhr_300 float %9.0g ldh_300 LDH (300 U/L)

creain_09 float %9.0g creain_09

Creatinina sérica (0.9 mg/dL)

creainr_09 float %9.0g creain_09

Creatinina sérica (0.9 mg/dL)

kalet byte %16.0g dNoSi Lopinavir/Ritonavir (LPV/r)

hcq byte %16.0g dNoSi Hidroxicloroquina

cloroq byte %16.0g dNoSi Cloroquina

tocil byte %16.0g dNoSi Tocilizumab

anaki byte %16.0g dNoSi Anakinra

remde byte %16.0g dNoSi Remdesivir

infb byte %16.0g dNoSi Interferón Beta-1B (IFNb)

colchi byte %16.0g dNoSi Colchicina

inmg byte %16.0g dNoSi Inmunoglobulina

baricit byte %16.0g dNoSi Baricitinib

beclo byte %16.0g dNoSi Beclometasona inhalada

corsis byte %16.0g dNoSi Corticoides sistémicos

fincor float %td Fecha inicio corticoides sistémicos

dinicor int %8.0g Días desde el inicio de los síntomas hasta el inicio de corticoides

sistémicos

dmacor float %9.0g Dosis máxima diaria de corticoides sistémicos (en mg de prednisona

equivalentes)

dcorsis byte %8.0g Días de tratamiento con corticoides sistémicos

docorac int %8.0g Dosis acumulada de corticoides durante el ingreso (en mg de prednisona

equivalen

dmegcor byte %8.0g Número de días con megadosis de corticoides (más de 150 mg equivalentes en

predn

aco byte %17.0g daco Anticoagulación oral durante el ingreso

hbmp byte %31.0g dhbmp Heparina de bajo peso molecular durante el ingreso

neum byte %16.0g dNoSi Neumonía bacteriana

sdra byte %16.0g dsdra Síndrome de distress respiratorio del adulto (SDRA)

icccom byte %16.0g dNoSi Insuficiencia cardiaca

arritmia float %16.0g dNoSi Arritmia cardiaca

arrit byte %16.0g darrit Arritmia cardiaca

iamcom byte %16.0g dNoSi Infarto de miocardio

miotis byte %16.0g dNoSi Miocarditis

comic byte %16.0g dNoSi Crisis comiciales

ictus float %16.0g dNoSi Ictus

ictcom byte %16.0g dictcom Ictus

irencom byte %16.0g dNoSi Insuficiencia renal aguda

etv byte %16.0g detv Enfermedad tromboembólica venosa

enfart byte %16.0g dNoSi Enfermedad arterial periférica aguda

sepsis byte %16.0g dNoSi Sepsis

cid byte %16.0g dNoSi Coagulación intravascular diseminada

shock byte %16.0g dNoSi Shock

fmo byte %16.0g dNoSi Fallo multiorgánico

gafalf byte %16.0g dNoSi Gafas nasales de alto flujo

vmni byte %16.0g dNoSi Ventilación mecánica no invasiva (VMNI)

dmvmni byte %8.0g Duración VMNI(días)

vmi byte %16.0g dNoSi Ventilación mecánica invasiva (VMI)

dvmi byte %8.0g Duración VMI (días)

prono byte %16.0g dNoSi ¿Posición prono?

uci byte %16.0g dNoSi Ingreso UCI

duci byte %8.0g Número de días de ingreso en UCI

------------------------------------------------------------------------------------------------------------------------

storage display value

variable name type format label variable label

------------------------------------------------------------------------------------------------------------------------

valido byte %42.0g dvalido ¿Válido?

dinicor_10 float %12.0g dinicor_10

Días de síntomas hasta inicio de corticoides

dmacor_125 float %10.0g dmacor_125

Dosis máxima diaria de corticoides

dcorsis_5 float %11.0g dcorsis_5

Días de tratamiento con corticoides

docorac_500 float %10.0g docorac_500

Dosis acumulada en el ingreso

dmegcor_3 float %25.0g dmegcor_3

Días con megadosis (Pulsos de GCC)

pulsos float %26.0g pulsos Uso de megadosis (Pulsos de GCC)

etev float %16.0g dNoSi Enf tromboembólica venosa

grupo float %12.0g grupo Corticosteroides

sdra_ byte %15.0g sdra_ Síndrome de distress respiratorio del adulto (SDRA)

------------------------------------------------------------------------------------------------------------------------

## Descriptive

### Numeric variables

----------------------------------------------------------------------------------------------------

Nº miss Mean Std. Dev. P25 P50 P75 Min Max Swilk

----------------------------------------------------------------------------------------------------

Días de estancia ho 14921 . 12,14 145 5 8 14 -3635 17294 0,000

Días de síntomas h 14760 161 5,396 142,6 3 7 9 -17285 369 0,000

Días desde al alta 565 14356 11,88 12,88 3 9 18 -60 74 0,000

Edad 14921 . 67,36 16,18 56,34 69,33 79,9 18,02 106 0,000

Peso (kg) 7154 7767 78,41 16,36 68 77 87 30 215 0,000

Índice de Charlson 14556 365 1,312 1,828 0 1 2 0 15 0,000

Charlson corregido p 14556 365 3,618 2,686 2 3 5 0 18 0,000

FEV1 (%) 490 14431 59,63 17,92 49 60 71 0 100 0,007

Saturación de oxíg 14512 409 92,89 5,966 91 94 97 40 100 0,000

FiO2 real si se disp 71 14850 59,1 34,32 21 60 100 21 100 0,004

pH en sangre arteria 7662 7259 7,433 ,1889 7,41 7,45 7,48 0 7,8 0,000

PCO2 al ingreso (mmH 7747 7174 35,56 8,808 30,7 34 39 0 193 0,000

PO2 al ingreso (mmHg 7405 7516 68,73 21,96 56 66 77,5 0 150 0,000

pO2/FiO2 al ingreso 7100 7821 286,9 98,82 233,3 288,6 342,4 0 714,3 0,000

FiO2 real si se disp 153 14768 43,39 32,65 21 21 70 21 100 0,000

Saturación de oxíg 12316 2605 94,21 5,32 93 95 97 40 100 0,000

FiO2 real si se disp 462 14459 74,93 24,06 55 80 100 21 100 0,000

pH en sangre arteria 3066 11855 7,384 ,4117 7,37 7,42 7,46 -1 7,69 0,000

PCO2 (mmHg) 3084 11837 42,08 11,91 35 40 46 -1 150 0,000

PO2 (mmHg) 2997 11924 78,28 27,96 60 73,2 90 -1 150 0,000

pO2/FiO2 (mmHg) (PO2 2813 12108 239 134,4 120 231,3 328,6 0 714,3 0,000

FiO2 real si se disp 422 14499 74,02 24,31 50 77,5 100 21 100 0,000

Hemoglobina (g/dL) 14817 104 13,7 1,893 12,6 13,9 15 3,7 20 0,000

Recuento de leucocit 14815 106 7376 5512 4790 6300 8510 5 90000 0,000

Recuento absoluto de 14746 175 5527 4599 3200 4600 6700 0 150000 0,000

Recuento absoluto de 14793 128 1168 2179 690 940 1300 0 90000 0,000

Plaquetas (x 10^6/L) 14813 108 206977 92631 148000 190000 247000 1000 1,4e+06 0,000

Proteína C reactiva 14295 626 87,15 88,82 19,05 60,1 127,7 0 1000 0,000

Procalcitonina (ng/m 7049 7872 ,4773 2,302 ,05 ,1 ,22 0 50 0,000

Ferritina sérica (m 5906 9015 939,4 1098 283 597 1215 0 9999 0,000

Dímero D (ng/mL) 11628 3293 1917 9624 380 670 1260 0 489000 0,000

Interleukina-6 (IL-6 1968 12953 68,62 176,3 11,55 29,8 65,05 0 3000 0,000

LDH (U/L) 12885 2036 371,7 223,8 246 321 432 3 9090 0,000

Límite superior nor 13107 1814 265,1 79,38 225 246 250 19 950 0,000

Creatinina sérica a 14778 143 1,103 ,8645 ,73 ,9 1,16 ,01 15 0,000

Urea (mg/dL) 11940 2981 48,32 37,6 27 37 55 10 496 0,000

Hemoglobina (g/dL) 13809 1112 12,88 1,796 11,8 13 14,1 2 20 0,000

Recuento de leucocit 13809 1112 7773 5683 4930 6510 9040 6 90000 0,000

Recuento absoluto de 13751 1170 5572 4627 2916 4380 6950 0 118000 0,000

Recuento absoluto de 13788 1133 1340 2565 700 1100 1600 0 97800 0,000

Plaquetas (x 10^6/L) 13794 1127 295049 135795 199000 276000 371000 1000 1,5e+06 0,000

Proteína C reactiva 13397 1524 58,79 82,33 7 23,8 74,9 0 960 0,000

Procalcitonina (ng/m 5376 9545 ,5594 2,86 ,05 ,09 ,2 0 50 0,000

Dímero D (ng/mL) 10637 4284 2356 8493 390 723 1489 0 315000 0,000

Interleukina-6 (IL-6 2269 12652 111,5 508,9 4,81 15,8 53,9 0 9999 0,000

LDH (U/L) 12339 2582 354,5 261,9 220 287 411 0 7605 0,000

Creatinina sérica ( 13758 1163 1,04 ,9301 ,68 ,82 1,05 0 15 0,000

Urea (mg/dL) 10768 4153 56,9 51,52 29 41 62 10 500 0,000

GOT-AST(U/L) 11638 3283 49,76 128,9 23 33 53 0 6383 0,000

GPT-ALT (U/L) 12939 1982 56,64 88,66 22 36 64 0 3142 0,000

Glucemia basal(mg/dL 13199 1722 120,2 57,96 87 100 133 18 832 0,000

Días desde el inici 5023 9898 11,51 9,689 7 10 14 0 131 0,000

Dosis máxima diaria 5040 9881 174,3 161,8 75 125 250 ,5 1500 0,000

Días de tratamiento 5074 9847 6,773 5,796 3 5 9 0 30 0,000

Dosis acumulada de c 4769 10152 674,9 608,5 300 510 937 0 5000 0,000

Número de días con 4794 10127 1,375 1,871 0 0 3 0 30 0,000

Duración VMNI(días 665 14256 4,662 4,387 2 3 6 0 35 0,000

Duración VMI (días 947 13974 14,05 10,47 7 11 19 0 60 0,000

Número de días de 1208 13713 15,36 12,4 6 12 21 0 83 0,000

----------------------------------------------------------------------------------------------------

Swilk: prueba de normalidad Shapiro-Wilk

### Categorical variables

The %valid excludes missing values in its calculation.

-----------------------------------------------------------------

Nº %tot Nº %val

-----------------------------------------------------------------

Motivo alta

14921 14921

0: 0. Mejoría: domicil 10993 73,67 10993 73,67

1: 1. Convalecencia: ce 869 5,82 869 5,82

2: 2. Exitus 3059 20,50 3059 20,50

-----------------------------------------------------------------

Exitus en el ingreso

14921 14921

0: 0. No 11862 79,50 11862 79,50

1: 1. Sí 3059 20,50 3059 20,50

-----------------------------------------------------------------

Causa del éxitus

14921 3012

0: 0. Infección por CO 2824 18,93 2824 93,76

1: 1. Otras 188 1,26 188 6,24

No disponible 47 0,31

-----------------------------------------------------------------

Reingreso (no incluir al paciente como nuevo si ha reingresado)

14921 14521

0: 0. No 13955 93,53 13955 96,10

1: 1. Sí 566 3,79 566 3,90

No disponible 400 2,68

-----------------------------------------------------------------

Causa del reingreso

14921 564

0: 0. COVID-19 220 1,47 220 39,01

1: 1. Otra 344 2,31 344 60,99

No disponible 2 0,01

-----------------------------------------------------------------

Motivo alta tras reingreso

14921 537

0: 0. Mejoría: domicil 435 2,92 435 81,01

1: 1. Convalecencia: ce 33 0,22 33 6,15

2: 2. Exitus 69 0,46 69 12,85

No disponible 29 0,19

-----------------------------------------------------------------

Fallecimiento durante el ingreso o reingreso

14921 14759

0: 0. No 11631 77,95 11631 78,81

1: 1. Sí 3128 20,96 3128 21,19

No disponible 162 1,09

-----------------------------------------------------------------

Causa del exitus tras reingreso

14921 68

0: 0. Infección por CO 29 0,19 29 42,65

1: 1. Otras 39 0,26 39 57,35

No disponible 1 0,01

-----------------------------------------------------------------

En caso de que el paciente no haya fallecido durante el ingreso o reingreso, ¿S

14921 12658

0: 0. No 8972 60,13 8972 70,88

1: 1. Sí 150 1,01 150 1,19

99: 99 3536 23,70 3536 27,93

No disponible 2263 15,17

-----------------------------------------------------------------

Resultado última PCR SARS-CoV-2

14921 12071

0: 0. Negativa 4879 32,70 4879 40,42

1: 1. Positiva 7192 48,20 7192 59,58

No disponible 2850 19,10

-----------------------------------------------------------------

Edad por décadas

14921 14921

1: 1. Menores de 40 añ 927 6,21 927 6,21

2: 2. Entre 40-50 años 1433 9,60 1433 9,60

3: 3. Entre 50-60 años 2385 15,98 2385 15,98

4: 4. Entre 60-70 años 2923 19,59 2923 19,59

5: 5. Entre 70-80 años 3570 23,93 3570 23,93

6: 6. Mayores de 80 añ 3683 24,68 3683 24,68

-----------------------------------------------------------------

Sexo

14921 14906

0: 0. Varón 8531 57,17 8531 57,23

1: 1. Mujer 6375 42,73 6375 42,77

No disponible 15 0,10

-----------------------------------------------------------------

-----------------------------------------------------------------

Nº %tot Nº %val

-----------------------------------------------------------------

Raza

14921 14678

0: 0. Caucásica 13254 88,83 13254 90,30

1: 1. Negra 54 0,36 54 0,37

2: 2. Latina 1182 7,92 1182 8,05

3: 3. Asiática 63 0,42 63 0,43

4: 4. Otras 125 0,84 125 0,85

No disponible 243 1,63

-----------------------------------------------------------------

Hipertensión arterial

14921 14899

0: 0. No 7326 49,10 7326 49,17

1: 1. Sí 7573 50,75 7573 50,83

No disponible 22 0,15

-----------------------------------------------------------------

Diabetes Mellitus

14921 14876

0: 0. No 12012 80,50 12012 80,75

1: 1. Sí 2864 19,19 2864 19,25

No disponible 45 0,30

-----------------------------------------------------------------

Diabetes sin lesión de órgano diana

14921 14890

0: 0. No 12793 85,74 12793 85,92

1: 1. Sí 2097 14,05 2097 14,08

No disponible 31 0,21

-----------------------------------------------------------------

Diabetes con lesión en órgano diana

14921 14889

0: 0. No 14098 94,48 14098 94,69

1: 1. Sí 791 5,30 791 5,31

No disponible 32 0,21

-----------------------------------------------------------------

Dislipemia

14921 14890

0: 0. No 8988 60,24 8988 60,36

1: 1. Sí 5902 39,55 5902 39,64

No disponible 31 0,21

-----------------------------------------------------------------

Obesidad (IMC mayor o igual a 30 kg/m2)

14921 13573

0: 0. No 10707 71,76 10707 78,88

1: 1. Sí 2866 19,21 2866 21,12

No disponible 1348 9,03

-----------------------------------------------------------------

Historia de tabaquismo

14921 14227

0: 0. Nunca ha fumado 9859 66,07 9859 69,30

1: 1. Exfumador 3613 24,21 3613 25,40

2: 2. Fumador 755 5,06 755 5,31

No disponible 694 4,65

-----------------------------------------------------------------

Enolismo

14921 14429

0: 0. No 13751 92,16 13751 95,30

1: 1. Sí 678 4,54 678 4,70

No disponible 492 3,30

-----------------------------------------------------------------

Fibrilación auricular

14921 14881

0: 0. No 13218 88,59 13218 88,82

1: 1. Sí 1663 11,15 1663 11,18

No disponible 40 0,27

-----------------------------------------------------------------

Insuficiencia cardiaca

14921 14893

0: 0. No 13822 92,63 13822 92,81

1: 1. Sí 1071 7,18 1071 7,19

No disponible 28 0,19

-----------------------------------------------------------------

Infarto de miocardio

14921 14899

0: 0. No 14021 93,97 14021 94,11

1: 1. Sí 878 5,88 878 5,89

No disponible 22 0,15

-----------------------------------------------------------------

-----------------------------------------------------------------

Nº %tot Nº %val

-----------------------------------------------------------------

Angina de pecho

14921 14895

0: 0. No 14370 96,31 14370 96,48

1: 1. Sí 525 3,52 525 3,52

No disponible 26 0,17

-----------------------------------------------------------------

Cardiopatía isquémica

14921 14886

0: 0. No 13698 91,80 13698 92,02

1: 1. Sí 1188 7,96 1188 7,98

No disponible 35 0,23

-----------------------------------------------------------------

EPOC

14921 14893

0: 0. No 13872 92,97 13872 93,14

1: 1. Sí 1021 6,84 1021 6,86

No disponible 28 0,19

-----------------------------------------------------------------

Bronquitis crónica

14921 14891

0: 0. No 14145 94,80 14145 94,99

1: 1. Sí 746 5,00 746 5,01

No disponible 30 0,20

-----------------------------------------------------------------

Asma

14921 14888

0: 0. No 13809 92,55 13809 92,75

1: 1. Sí 1079 7,23 1079 7,25

No disponible 33 0,22

-----------------------------------------------------------------

Síndrome de apnea-hipopnea del sueño

14921 14825

0: 0. No 13941 93,43 13941 94,04

1: 1. Sí 884 5,92 884 5,96

No disponible 96 0,64

-----------------------------------------------------------------

Ictus/AIT

14921 14873

0: 0. No 13792 92,43 13792 92,73

1: 1. Sí 1081 7,24 1081 7,27

No disponible 48 0,32

-----------------------------------------------------------------

Accidente isquémico transitorio o ictus isquémico sin secuelas

14921 14885

0: 0. No 14194 95,13 14194 95,36

1: 1. Sí 691 4,63 691 4,64

No disponible 36 0,24

-----------------------------------------------------------------

ACV isquémico o hemorrágico con secuelas

14921 14896

0: 0. No 14471 96,98 14471 97,15

1: 1. Sí 425 2,85 425 2,85

No disponible 25 0,17

-----------------------------------------------------------------

Hemiplejia o paraplejia

14921 14898

0: 0. No 14653 98,20 14653 98,36

1: 1. Sí 245 1,64 245 1,64

No disponible 23 0,15

-----------------------------------------------------------------

Demencia

14921 14890

0: 0. No 13394 89,77 13394 89,95

1: 1. Sí 1496 10,03 1496 10,05

No disponible 31 0,21

-----------------------------------------------------------------

Enfermedad neurológica degenerativa

14921 14897

0: 0. No 13541 90,75 13541 90,90

1: 1. Sí 1356 9,09 1356 9,10

No disponible 24 0,16

-----------------------------------------------------------------

Hepatopatía crónica

14921 14860

0: 0. No 14308 95,89 14308 96,29

1: 1. Sí 552 3,70 552 3,71

No disponible 61 0,41

-----------------------------------------------------------------

-----------------------------------------------------------------

Nº %tot Nº %val

-----------------------------------------------------------------

Hepatopatía crónica leve

14921 14881

0: 0. No 14467 96,96 14467 97,22

1: 1. Sí 414 2,77 414 2,78

No disponible 40 0,27

-----------------------------------------------------------------

Hepatopatía crónica moderada-severa

14921 14887

0: 0. No 14739 98,78 14739 99,01

1: 1. Sí 148 0,99 148 0,99

No disponible 34 0,23

-----------------------------------------------------------------

Insuficiencia renal crónica moderada-severa

14921 14888

0: 0. No 13984 93,72 13984 93,93

1: 1. Sí 904 6,06 904 6,07

No disponible 33 0,22

-----------------------------------------------------------------

Paciente habitualmente en diálisis

14921 14865

0: 0. No 14692 98,47 14692 98,84

1: 1. Hemodiálisis 152 1,02 152 1,02

2: 2. Diálisis periton 21 0,14 21 0,14

No disponible 56 0,38

-----------------------------------------------------------------

Enfermedad vascular periférica

14921 14885

0: 0. No 14185 95,07 14185 95,30

1: 1. Sí 700 4,69 700 4,70

No disponible 36 0,24

-----------------------------------------------------------------

Úlcera gastroduodenal

14921 14883

0: 0. No 14496 97,15 14496 97,40

1: 1. Sí 387 2,59 387 2,60

No disponible 38 0,25

-----------------------------------------------------------------

Cáncer

14921 14878

0: 0. No 13637 91,39 13637 91,66

1: 1. Sí 1241 8,32 1241 8,34

No disponible 43 0,29

-----------------------------------------------------------------

Neoplasia sólida sin metástasis

14921 14900

0: 0. No 13968 93,61 13968 93,74

1: 1. Sí 932 6,25 932 6,26

No disponible 21 0,14

-----------------------------------------------------------------

Neoplasia sólida con metástasis

14921 14888

0: 0. No 14565 97,61 14565 97,83

1: 1. Sí 323 2,16 323 2,17

No disponible 33 0,22

-----------------------------------------------------------------

Leucemia

14921 14903

0: 0. No 14724 98,68 14724 98,80

1: 1. Sí 179 1,20 179 1,20

No disponible 18 0,12

-----------------------------------------------------------------

Linfoma

14921 14892

0: 0. No 14680 98,38 14680 98,58

1: 1. Sí 212 1,42 212 1,42

No disponible 29 0,19

-----------------------------------------------------------------

Enfermedad del tejido conectivo

14921 14883

0: 0. No 14531 97,39 14531 97,63

1: 1. Sí 352 2,36 352 2,37

No disponible 38 0,25

-----------------------------------------------------------------

Paciente con enfermedad rara/minoritaria

14921 14859

0: 0. No 14530 97,38 14530 97,79

1: 1. Sí 329 2,20 329 2,21

No disponible 62 0,42

-----------------------------------------------------------------

-----------------------------------------------------------------

Nº %tot Nº %val

-----------------------------------------------------------------

Trastorno de ansiedad

14921 14865

0: 0. No 13712 91,90 13712 92,24

1: 1. Sí 1153 7,73 1153 7,76

No disponible 56 0,38

-----------------------------------------------------------------

Depresión

14921 14863

0: 0. No 13327 89,32 13327 89,67

1: 1. Sí 1536 10,29 1536 10,33

No disponible 58 0,39

-----------------------------------------------------------------

SIDA (con criterios definitorios)

14921 14866

0: 0. No 14819 99,32 14819 99,68

1: 1. Sí 47 0,31 47 0,32

No disponible 55 0,37

-----------------------------------------------------------------

Infección VIH conocida previa (con o sin criterios de SIDA)

14921 14861

0: 0. No 14759 98,91 14759 99,31

1: 1. Sí 102 0,68 102 0,69

No disponible 60 0,40

-----------------------------------------------------------------

Inmunodepresores tto habitual

14921 14874

0: 0. No 14344 96,13 14344 96,44

1: 1. Sí 530 3,55 530 3,56

No disponible 47 0,31

-----------------------------------------------------------------

Tratamiento habitual con inmunosupresores (si está con varios, elegir otros e in

14921 14874

0: 0. No 14344 96,13 14344 96,44

1: 1. Azatioprina 63 0,42 63 0,42

2: 2. Metotrexato 126 0,84 126 0,85

3: 3. Tacrolimus 27 0,18 27 0,18

4: 4. Ciclosporina 8 0,05 8 0,05

5: 5. Ciclofosfamida 10 0,07 10 0,07

6: 6. Micofenolato 29 0,19 29 0,19

7: 7. Otros (especifica 267 1,79 267 1,80

No disponible 47 0,31

-----------------------------------------------------------------

Rapamicina (Sirolimus)

14921 14857

0: 0. No 14812 99,27 14812 99,70

1: 1. Sí 45 0,30 45 0,30

No disponible 64 0,43

-----------------------------------------------------------------

Terapias biológicas de base (anticuerpos monoclonales)

14921 14887

0: 0. No 14691 98,46 14691 98,68

1: 1. Sí 196 1,31 196 1,32

No disponible 34 0,23

-----------------------------------------------------------------

Tratamiento habitual con corticoides sistémicos

14921 14885

0: 0. No 14232 95,38 14232 95,61

1: 1. Sí 653 4,38 653 4,39

No disponible 36 0,24

-----------------------------------------------------------------

Tratamiento habitual con corticoides inhalados

14921 14843

0: 0. No 13452 90,15 13452 90,63

1: 1. Sí 1391 9,32 1391 9,37

No disponible 78 0,52

-----------------------------------------------------------------

SatO2

14921 14512

0: 0. <90% 3307 22,16 3307 22,79

1: 1. >90% 11205 75,10 11205 77,21

No disponible 409 2,74

-----------------------------------------------------------------

-----------------------------------------------------------------

Nº %tot Nº %val

-----------------------------------------------------------------

FiO2 con la que se ha obtenido la SpO2

14921 14244

0: 0. Aire ambiente 11434 76,63 11434 80,27

1: 1. Gafas nasales 1 l 86 0,58 86 0,60

2: 2. gafas nasales 2 l 1130 7,57 1130 7,93

3: 3. Gafas nasales 3 l 381 2,55 381 2,67

4: 4. Gafas nasales 4 l 225 1,51 225 1,58

5: 5. Gafas nasales 5 l 61 0,41 61 0,43

6: 6. Ventimask 24% 39 0,26 39 0,27

7: 7. Ventimask 28% 71 0,48 71 0,50

8: 8. Ventimask 30% 72 0,48 72 0,51

9: 9. Ventimask 35% 94 0,63 94 0,66

10: 10. Ventimask 40% 73 0,49 73 0,51

11: 11. Ventimask 50% 124 0,83 124 0,87

12: 12. Reservorio (80%) 382 2,56 382 2,68

13: 13. FiO2 real si se 72 0,48 72 0,51

No disponible 677 4,54

-----------------------------------------------------------------

FiO2 con la que se ha obtenido la gasometría

14921 7604

0: 0. Aire ambiente 5470 36,66 5470 71,94

1: 1. Gafas nasales 1 l 47 0,31 47 0,62

2: 2. gafas nasales 2 l 766 5,13 766 10,07

3: 3. Gafas nasales 3 l 225 1,51 225 2,96

4: 4. Gafas nasales 4 l 144 0,97 144 1,89

5: 5. Gafas nasales 5 l 43 0,29 43 0,57

6: 6. Ventimask 24% 45 0,30 45 0,59

7: 7. Ventimask 28% 70 0,47 70 0,92

8: 8. Ventimask 30% 56 0,38 56 0,74

9: 9. Ventimask 35% 87 0,58 87 1,14

10: 10. Ventimask 40% 57 0,38 57 0,75

11: 11. Ventimask 50% 118 0,79 118 1,55

12: 12. Reservorio (80%) 321 2,15 321 4,22

13: 13. FiO2 real si se 155 1,04 155 2,04

No disponible 7317 49,04

-----------------------------------------------------------------

SatO2 posterior

14921 12316

0: 0. <90% 1642 11,00 1642 13,33

1: 1. >90% 10674 71,54 10674 86,67

No disponible 2605 17,46

-----------------------------------------------------------------

FiO2 con la que se ha obtenido la SpO2

14921 12113

0: 0. Aire ambiente 6420 43,03 6420 53,00

1: 1. Gafas nasales 1 l 273 1,83 273 2,25

2: 2. gafas nasales 2 l 1447 9,70 1447 11,95

3: 3. Gafas nasales 3 l 495 3,32 495 4,09

4: 4. Gafas nasales 4 l 363 2,43 363 3,00

5: 5. Gafas nasales 5 l 143 0,96 143 1,18

6: 6. Ventimask 24% 37 0,25 37 0,31

7: 7. Ventimask 28% 85 0,57 85 0,70

8: 8. Ventimask 30% 99 0,66 99 0,82

9: 9. Ventimask 35% 144 0,97 144 1,19

10: 10. Ventimask 40% 157 1,05 157 1,30

11: 11. Ventimask 50% 328 2,20 328 2,71

12: 12. Reservorio (80%) 1653 11,08 1653 13,65

13: 13. FiO2 real si se 469 3,14 469 3,87

No disponible 2808 18,82

-----------------------------------------------------------------

FiO2 con la que se ha obtenido la gasometría

14921 3160

0: 0. Aire ambiente 1198 8,03 1198 37,91

1: 1. Gafas nasales 1 l 54 0,36 54 1,71

2: 2. gafas nasales 2 l 352 2,36 352 11,14

3: 3. Gafas nasales 3 l 115 0,77 115 3,64

4: 4. Gafas nasales 4 l 91 0,61 91 2,88

5: 5. Gafas nasales 5 l 27 0,18 27 0,85

6: 6. Ventimask 24% 11 0,07 11 0,35

7: 7. Ventimask 28% 36 0,24 36 1,14

8: 8. Ventimask 30% 30 0,20 30 0,95

9: 9. Ventimask 35% 48 0,32 48 1,52

10: 10. Ventimask 40% 63 0,42 63 1,99

11: 11. Ventimask 50% 156 1,05 156 4,94

12: 12. Reservorio (80%) 553 3,71 553 17,50

13: 13. FiO2 real si se 426 2,86 426 13,48

No disponible 11761 78,82

-----------------------------------------------------------------

-----------------------------------------------------------------

Nº %tot Nº %val

-----------------------------------------------------------------

Condensación

14921 14739

0: 0. No 7536 50,51 7536 51,13

1: 1. Unilateral 2581 17,30 2581 17,51

2: 2. Bilateral 4622 30,98 4622 31,36

No disponible 182 1,22

-----------------------------------------------------------------

Infiltrado intersticial/vidrio deslustrado

14921 14746

0: 0. No 5509 36,92 5509 37,36

1: 1. Unilateral 1526 10,23 1526 10,35

2: 2. Bilateral 7711 51,68 7711 52,29

No disponible 175 1,17

-----------------------------------------------------------------

Derrame pleural

14921 14738

0: 0. No 14054 94,19 14054 95,36

1: 1. Unilateral 451 3,02 451 3,06

2: 2. Bilateral 233 1,56 233 1,58

No disponible 183 1,23

-----------------------------------------------------------------

¿Realizada TC torácica?

14921 14793

0: 0. No 13915 93,26 13915 94,06

1: 1. Sí 878 5,88 878 5,94

No disponible 128 0,86

-----------------------------------------------------------------

Condensación

14921 11113

0: 0. No 5159 34,58 5159 46,42

1: 1. Unilateral 1559 10,45 1559 14,03

2: 2. Bilateral 4395 29,46 4395 39,55

No disponible 3808 25,52

-----------------------------------------------------------------

Infiltrado intersticial/vidrio deslustrado

14921 11104

0: 0. No 3909 26,20 3909 35,20

1: 1. Unilateral 825 5,53 825 7,43

2: 2. Bilateral 6370 42,69 6370 57,37

No disponible 3817 25,58

-----------------------------------------------------------------

Derrame pleural

14921 11092

0: 0. No 10576 70,88 10576 95,35

1: 1. Unilateral 339 2,27 339 3,06

2: 2. Bilateral 177 1,19 177 1,60

No disponible 3829 25,66

-----------------------------------------------------------------

¿Empeoramiento radiológico?

14921 11135

0: 0. No 6738 45,16 6738 60,51

1: 1. Sí 4397 29,47 4397 39,49

No disponible 3786 25,37

-----------------------------------------------------------------

Condensación al ingreso

14921 14739

0: 0. No 7536 50,51 7536 51,13

1: 1. Sí 7203 48,27 7203 48,87

No disponible 182 1,22

-----------------------------------------------------------------

Infiltrado intesticial al ingreso

14921 14746

0: 0. No 5509 36,92 5509 37,36

1: 1. Sí 9237 61,91 9237 62,64

No disponible 175 1,17

-----------------------------------------------------------------

Derrame pleural al ingreso

14921 14738

0: 0. No 14054 94,19 14054 95,36

1: 1. Sí 684 4,58 684 4,64

No disponible 183 1,23

-----------------------------------------------------------------

Condensación en la evolución

14921 11113

0: 0. No 5159 34,58 5159 46,42

1: 1. Sí 5954 39,90 5954 53,58

No disponible 3808 25,52

-----------------------------------------------------------------

-----------------------------------------------------------------

Nº %tot Nº %val

-----------------------------------------------------------------

Infiltrado intesticial en la evolución

14921 11104

0: 0. No 3909 26,20 3909 35,20

1: 1. Sí 7195 48,22 7195 64,80

No disponible 3817 25,58

-----------------------------------------------------------------

Derrame pleural en la evolución

14921 11092

0: 0. No 10576 70,88 10576 95,35

1: 1. Sí 516 3,46 516 4,65

No disponible 3829 25,66

-----------------------------------------------------------------

Hemoglobina (12 g/dL)

14921 14817

1: 1. <12 2594 17,38 2594 17,51

2: 2. >12 12223 81,92 12223 82,49

No disponible 104 0,70

-----------------------------------------------------------------

Hemoglobina (12 g/dL)

14921 13809

1: 1. <12 4133 27,70 4133 29,93

2: 2. >12 9676 64,85 9676 70,07

No disponible 1112 7,45

-----------------------------------------------------------------

Leucocitosis (10 x 10^6/L)

14921 14815

1: 1. <10.000 12531 83,98 12531 84,58

2: 2. >10.000 2284 15,31 2284 15,42

No disponible 106 0,71

-----------------------------------------------------------------

Leucocitos (4 x 10^6/L)

14921 14815

1: 1. <4.000 2046 13,71 2046 13,81

2: 2. >4.000 12769 85,58 12769 86,19

No disponible 106 0,71

-----------------------------------------------------------------

Leucocitosis (10 x 10^6/L)

14921 13809

1: 1. <10.000 11146 74,70 11146 80,72

2: 2. >10.000 2663 17,85 2663 19,28

No disponible 1112 7,45

-----------------------------------------------------------------

Leucocitos (4 x 10^6/L)

14921 13809

1: 1. <4.000 1610 10,79 1610 11,66

2: 2. >4.000 12199 81,76 12199 88,34

No disponible 1112 7,45

-----------------------------------------------------------------

Linfocitos (1.3 x 10^6/L)

14921 14793

1: 1. <1300 11259 75,46 11259 76,11

2: 2. >1300 3534 23,68 3534 23,89

No disponible 128 0,86

-----------------------------------------------------------------

Linfocitos (1.3 x 10^6/L)

14921 13788

1: 1. <1300 8573 57,46 8573 62,18

2: 2. >1300 5215 34,95 5215 37,82

No disponible 1133 7,59

-----------------------------------------------------------------

Plaquetas (180 x 10^6/L)

14921 14813

1: 1. <180 6601 44,24 6601 44,56

2: 2. >180 8212 55,04 8212 55,44

No disponible 108 0,72

-----------------------------------------------------------------

Plaquetas (180 x 10^6/L)

14921 13794

1: 1. <180 2655 17,79 2655 19,25

2: 2. >180 11139 74,65 11139 80,75

No disponible 1127 7,55

-----------------------------------------------------------------

PCR (50 mg/L)

14921 14295

1: 1. <50 (<5 mg/dL) 6470 43,36 6470 45,26

2: 2. >50 (>5 mg/dL) 7825 52,44 7825 54,74

No disponible 626 4,20

-----------------------------------------------------------------

-----------------------------------------------------------------

Nº %tot Nº %val

-----------------------------------------------------------------

PCR (50 mg/L)

14921 13397

1: 1. <50 (<5 mg/dL) 8941 59,92 8941 66,74

2: 2. >50 (>5 mg/dL) 4456 29,86 4456 33,26

No disponible 1524 10,21

-----------------------------------------------------------------

Procalcitonina (0.5 ng/mL)

14921 7049

1: 1. <0.5 6207 41,60 6207 88,06

2: 2. >0.5 842 5,64 842 11,94

No disponible 7872 52,76

-----------------------------------------------------------------

Procalcitonina (0.5 ng/mL)

14921 5376

1: 1. <0.5 4701 31,51 4701 87,44

2: 2. >0.5 675 4,52 675 12,56

No disponible 9545 63,97

-----------------------------------------------------------------

Ferritina (1000 mcg/L)

14921 5906

1: 1. <1000 4040 27,08 4040 68,41

2: 2. >1000 1866 12,51 1866 31,59

No disponible 9015 60,42

-----------------------------------------------------------------

Ferritina (274 mcg/L)

14921 5906

1: 1. <274 1431 9,59 1431 24,23

2: 2. >274 4475 29,99 4475 75,77

No disponible 9015 60,42

-----------------------------------------------------------------

Dímero D (250 ng/mL)

14921 11627

1: 1. <250 1457 9,76 1457 12,53

2: 2. >250 10170 68,16 10170 87,47

No disponible 3294 22,08

-----------------------------------------------------------------

Dímero D (250 ng/mL)

14921 10637

1: 1. <250 1328 8,90 1328 12,48

2: 2. >250 9309 62,39 9309 87,52

No disponible 4284 28,71

-----------------------------------------------------------------

IL6 (4.3 pg/mL)

14921 1968

1: 1. <4.3 230 1,54 230 11,69

2: 2. >4.3 1738 11,65 1738 88,31

No disponible 12953 86,81

-----------------------------------------------------------------

IL6 (4.3 pg/mL)

14921 2269

1: 1. <4.3 522 3,50 522 23,01

2: 2. >4.3 1747 11,71 1747 76,99

No disponible 12652 84,79

-----------------------------------------------------------------

LDH (300 U/L)

14921 12885

1: 1. <300 5626 37,71 5626 43,66

2: 2. >300 7259 48,65 7259 56,34

No disponible 2036 13,65

-----------------------------------------------------------------

LDH (300 U/L)

14921 12339

1: 1. <300 6704 44,93 6704 54,33

2: 2. >300 5635 37,77 5635 45,67

No disponible 2582 17,30

-----------------------------------------------------------------

Creatinina sérica (0.9 mg/dL)

14921 14778

1: 1. <0.9 7499 50,26 7499 50,74

2: 2. >0.9 7279 48,78 7279 49,26

No disponible 143 0,96

-----------------------------------------------------------------

Creatinina sérica (0.9 mg/dL)

14921 13758

1: 1. <0.9 8462 56,71 8462 61,51

2: 2. >0.9 5296 35,49 5296 38,49

No disponible 1163 7,79

-----------------------------------------------------------------

-----------------------------------------------------------------

Nº %tot Nº %val

-----------------------------------------------------------------

Lopinavir/Ritonavir (LPV/r)

14921 14901

0: 0. No 5753 38,56 5753 38,61

1: 1. Sí 9148 61,31 9148 61,39

No disponible 20 0,13

-----------------------------------------------------------------

Hidroxicloroquina

14921 14911

0: 0. No 2139 14,34 2139 14,35

1: 1. Sí 12772 85,60 12772 85,65

No disponible 10 0,07

-----------------------------------------------------------------

Cloroquina

14921 14867

0: 0. No 14196 95,14 14196 95,49

1: 1. Sí 671 4,50 671 4,51

No disponible 54 0,36

-----------------------------------------------------------------

Tocilizumab

14921 14882

0: 0. No 13625 91,31 13625 91,55

1: 1. Sí 1257 8,42 1257 8,45

No disponible 39 0,26

-----------------------------------------------------------------

Anakinra

14921 14787

0: 0. No 14697 98,50 14697 99,39

1: 1. Sí 90 0,60 90 0,61

No disponible 134 0,90

-----------------------------------------------------------------

Remdesivir

14921 14810

0: 0. No 14743 98,81 14743 99,55

1: 1. Sí 67 0,45 67 0,45

No disponible 111 0,74

-----------------------------------------------------------------

Interferón Beta-1B (IFNb)

14921 14844

0: 0. No 13183 88,35 13183 88,81

1: 1. Sí 1661 11,13 1661 11,19

No disponible 77 0,52

-----------------------------------------------------------------

Colchicina

14921 14654

0: 0. No 14520 97,31 14520 99,09

1: 1. Sí 134 0,90 134 0,91

No disponible 267 1,79

-----------------------------------------------------------------

Inmunoglobulina

14921 14666

0: 0. No 14597 97,83 14597 99,53

1: 1. Sí 69 0,46 69 0,47

No disponible 255 1,71

-----------------------------------------------------------------

Baricitinib

14921 11840

0: 0. No 11748 78,73 11748 99,22

1: 1. Sí 92 0,62 92 0,78

No disponible 3081 20,65

-----------------------------------------------------------------

Beclometasona inhalada

14921 14737

0: 0. No 13975 93,66 13975 94,83

1: 1. Sí 762 5,11 762 5,17

No disponible 184 1,23

-----------------------------------------------------------------

Corticoides sistémicos

14921 14921

0: 0. No 9659 64,73 9659 64,73

1: 1. Sí 5262 35,27 5262 35,27

-----------------------------------------------------------------

Anticoagulación oral durante el ingreso

14921 14852

0: 0. No 14329 96,03 14329 96,48

1: 1. Antivitamina K 253 1,70 253 1,70

2: 2. ACOD 270 1,81 270 1,82

No disponible 69 0,46

-----------------------------------------------------------------

-----------------------------------------------------------------

Nº %tot Nº %val

-----------------------------------------------------------------

Heparina de bajo peso molecular durante el ingreso

14921 14846

0: 0. No 2605 17,46 2605 17,55

1: 1. Dosis profilácti 9619 64,47 9619 64,79

2: 2. Dosis plenas anti 1628 10,91 1628 10,97

3: 3. Dosis intermedias 994 6,66 994 6,70

No disponible 75 0,50

-----------------------------------------------------------------

Neumonía bacteriana

14921 14877

0: 0. No 13223 88,62 13223 88,88

1: 1. Sí 1654 11,09 1654 11,12

No disponible 44 0,29

-----------------------------------------------------------------

Síndrome de distress respiratorio del adulto (SDRA)

14921 14857

0: 0. No 9961 66,76 9961 67,05

1: 1. Leve 1185 7,94 1185 7,98

2: 2. Moderado 1080 7,24 1080 7,27

3: 3. Severo 2631 17,63 2631 17,71

No disponible 64 0,43

-----------------------------------------------------------------

Síndrome de distress respiratorio del adulto (SDRA)

14921 14857

0: No/Leve 11146 74,70 11146 75,02

1: Moderado/Severo 3711 24,87 3711 24,98

No disponible 64 0,43

-----------------------------------------------------------------

Insuficiencia cardiaca

14921 14888

0: 0. No 14026 94,00 14026 94,21

1: 1. Sí 862 5,78 862 5,79

No disponible 33 0,22

-----------------------------------------------------------------

Arritmia cardiaca

14921 14878

0: 0. No 14306 95,88 14306 96,16

1: 1. Sí 572 3,83 572 3,84

No disponible 43 0,29

-----------------------------------------------------------------

Arritmia cardiaca

14921 14878

0: 0. No 14306 95,88 14306 96,16

1: 1. Auriculares 511 3,42 511 3,43

2: 2. Ventriculares 40 0,27 40 0,27

3: 3. Ambas 21 0,14 21 0,14

No disponible 43 0,29

-----------------------------------------------------------------

Infarto de miocardio

14921 14883

0: 0. No 14760 98,92 14760 99,17

1: 1. Sí 123 0,82 123 0,83

No disponible 38 0,25

-----------------------------------------------------------------

Miocarditis

14921 14886

0: 0. No 14751 98,86 14751 99,09

1: 1. Sí 135 0,90 135 0,91

No disponible 35 0,23

-----------------------------------------------------------------

Crisis comiciales

14921 14891

0: 0. No 14796 99,16 14796 99,36

1: 1. Sí 95 0,64 95 0,64

No disponible 30 0,20

-----------------------------------------------------------------

Ictus

14921 14882

0: 0. No 14778 99,04 14778 99,30

1: 1. Sí 104 0,70 104 0,70

No disponible 39 0,26

-----------------------------------------------------------------

Ictus

14921 14882

0: 0. No 14778 99,04 14778 99,30

1: 1. Isquémico 93 0,62 93 0,62

2: 2. Hemorrágico 11 0,07 11 0,07

No disponible 39 0,26

-----------------------------------------------------------------

-----------------------------------------------------------------

Nº %tot Nº %val

-----------------------------------------------------------------

Insuficiencia renal aguda

14921 14882

0: 0. No 12800 85,79 12800 86,01

1: 1. Sí 2082 13,95 2082 13,99

No disponible 39 0,26

-----------------------------------------------------------------

Enfermedad tromboembólica venosa

14921 14862

0: 0. No 14545 97,48 14545 97,87

1: 1. TVP 79 0,53 79 0,53

2: 2. TEP 216 1,45 216 1,45

3: 3. TVP+TEP 22 0,15 22 0,15

No disponible 59 0,40

-----------------------------------------------------------------

Enfermedad arterial periférica aguda

14921 14811

0: 0. No 14734 98,75 14734 99,48

1: 1. Sí 77 0,52 77 0,52

No disponible 110 0,74

-----------------------------------------------------------------

Sepsis

14921 14880

0: 0. No 13962 93,57 13962 93,83

1: 1. Sí 918 6,15 918 6,17

No disponible 41 0,27

-----------------------------------------------------------------

Coagulación intravascular diseminada

14921 14864

0: 0. No 14696 98,49 14696 98,87

1: 1. Sí 168 1,13 168 1,13

No disponible 57 0,38

-----------------------------------------------------------------

Shock

14921 14863

0: 0. No 14187 95,08 14187 95,45

1: 1. Sí 676 4,53 676 4,55

No disponible 58 0,39

-----------------------------------------------------------------

Fallo multiorgánico

14921 14872

0: 0. No 13965 93,59 13965 93,90

1: 1. Sí 907 6,08 907 6,10

No disponible 49 0,33

-----------------------------------------------------------------

Gafas nasales de alto flujo

14921 14811

0: 0. No 13622 91,29 13622 91,97

1: 1. Sí 1189 7,97 1189 8,03

No disponible 110 0,74

-----------------------------------------------------------------

Ventilación mecánica no invasiva (VMNI)

14921 14872

0: 0. No 14153 94,85 14153 95,17

1: 1. Sí 719 4,82 719 4,83

No disponible 49 0,33

-----------------------------------------------------------------

Ventilación mecánica invasiva (VMI)

14921 14879

0: 0. No 13904 93,18 13904 93,45

1: 1. Sí 975 6,53 975 6,55

No disponible 42 0,28

-----------------------------------------------------------------

¿Posición prono?

14921 14860

0: 0. No 13341 89,41 13341 89,78

1: 1. Sí 1519 10,18 1519 10,22

No disponible 61 0,41

-----------------------------------------------------------------

Ingreso UCI

14921 14912

0: 0. No 13694 91,78 13694 91,83

1: 1. Sí 1218 8,16 1218 8,17

No disponible 9 0,06

-----------------------------------------------------------------

-----------------------------------------------------------------

Nº %tot Nº %val

-----------------------------------------------------------------

¿Válido?

14921 14921

0: 0. No 2218 14,86 2218 14,86

1: 1. Sí, completo 3429 22,98 3429 22,98

2: 2. Sí, con algunas 9274 62,15 9274 62,15

-----------------------------------------------------------------

Días de síntomas hasta inicio de corticoides

14921 5023

1: 1. <10 días 2719 18,22 2719 54,13

2: 2. >10 días 2304 15,44 2304 45,87

No disponible 9898 66,34

-----------------------------------------------------------------

Dosis máxima diaria de corticoides

14921 5040

1: 1. <125 mg 2716 18,20 2716 53,89

2: 2. >125 mg 2324 15,58 2324 46,11

No disponible 9881 66,22

-----------------------------------------------------------------

Días de tratamiento con corticoides

14921 5074

1: 1. <5 días 2902 19,45 2902 57,19

2: 2. >5 días 2172 14,56 2172 42,81

No disponible 9847 65,99

-----------------------------------------------------------------

Dosis acumulada en el ingreso

14921 4769

1: 1. <500 mg 2380 15,95 2380 49,91

2: 2. >500 mg 2389 16,01 2389 50,09

No disponible 10152 68,04

-----------------------------------------------------------------

Días con megadosis (Pulsos de GCC)

14921 4794

0: 0. No han recibido p 2578 17,28 2578 53,78

1: 1. <3 días 1794 12,02 1794 37,42

2: 2. >3 días 422 2,83 422 8,80

No disponible 10127 67,87

-----------------------------------------------------------------

Uso de megadosis (Pulsos de GCC)

14921 4794

0: 0. No han recibido p 2578 17,28 2578 53,78

1: 1. Sí han recibido 2216 14,85 2216 46,22

No disponible 10127 67,87

-----------------------------------------------------------------

Enf tromboembólica venosa

14921 14862

0: 0. No 14545 97,48 14545 97,87

1: 1. Sí 317 2,12 317 2,13

No disponible 59 0,40

-----------------------------------------------------------------

## Bivariante tablas 4 y 5

### Variables categóricas

----------------------------------------------------------------------------------------

Corticosteroides

A:No CS

B:Low-dose CS

C:CS megadoses

----------------------------------------------------------------------------------------

Condensación al ingreso p(ji2)= 0,000

A B C p(AB) p(AC) p(BC)

0: 0. No 5235 52,4% 1246 49,0% 1055 47,8% 0,002 0,000 0,432

1: 1. Sí 4753 47,6% 1298 51,0% 1152 52,2% 0,002 0,000 0,432

TOTAL 9988 2544 2207 0,002 0,000 0,419

----------------------------------------------------------------------------------------

Infiltrado intesticial al ingreso p(ji2)= 0,000

A B C p(AB) p(AC) p(BC)

0: 0. No 3939 39,4% 935 36,6% 635 28,8% 0,010 0,000 0,000

1: 1. Sí 6050 60,6% 1617 63,4% 1570 71,2% 0,010 0,000 0,000

TOTAL 9989 2552 2205 0,010 0,000 0,000

----------------------------------------------------------------------------------------

Derrame pleural al ingreso p(ji2)= 0,085

A B C p(AB) p(AC) p(BC)

0: 0. No 9521 95,4% 2416 94,6% 2117 96,0% 0,106 0,280 0,034

1: 1. Sí 458 4,6% 137 5,4% 89 4,0% 0,106 0,280 0,034

TOTAL 9979 2553 2206 0,100 0,254 0,031

----------------------------------------------------------------------------------------

Hemoglobina (12 g/dL) p(ji2)= 0,000

A B C p(AB) p(AC) p(BC)

1: 1. <12 1690 16,8% 551 21,5% 353 16,0% 0,000 0,344 0,000

2: 2. >12 8352 83,2% 2015 78,5% 1856 84,0% 0,000 0,344 0,000

TOTAL 10042 2566 2209 0,000 0,332 0,000

----------------------------------------------------------------------------------------

Plaquetas (180 x 10^6/L) p(ji2)= 0,029

A B C p(AB) p(AC) p(BC)

1: 1. <180 4412 43,9% 1200 46,8% 989 44,9% 0,009 0,407 0,190

2: 2. >180 5635 56,1% 1363 53,2% 1214 55,1% 0,009 0,407 0,190

TOTAL 10047 2563 2203 0,008 0,402 0,183

----------------------------------------------------------------------------------------

Leucocitosis (10 x 10^6/L) p(ji2)= 0,000

A B C p(AB) p(AC) p(BC)

1: 1. <10.000 8717 86,8% 2031 79,2% 1783 80,7% 0,000 0,000 0,181

2: 2. >10.000 1323 13,2% 535 20,8% 426 19,3% 0,000 0,000 0,181

TOTAL 10040 2566 2209 0,000 0,000 0,179

----------------------------------------------------------------------------------------

Leucocitos (4 x 10^6/L) p(ji2)= 0,001

A B C p(AB) p(AC) p(BC)

1: 1. <4.000 1458 14,5% 325 12,7% 263 11,9% 0,016 0,001 0,453

2: 2. >4.000 8582 85,5% 2241 87,3% 1946 88,1% 0,016 0,001 0,453

TOTAL 10040 2566 2209 0,016 0,001 0,426

----------------------------------------------------------------------------------------

Linfocitos (1.3 x 10^6/L) p(ji2)= 0,000

A B C p(AB) p(AC) p(BC)

1: 1. <1300 7378 73,6% 2070 80,8% 1811 82,1% 0,000 0,000 0,279

2: 2. >1300 2648 26,4% 491 19,2% 395 17,9% 0,000 0,000 0,279

TOTAL 10026 2561 2206 0,000 0,000 0,262

----------------------------------------------------------------------------------------

PCR (50 mg/L) p(ji2)= 0,000

A B C p(AB) p(AC) p(BC)

1: 1. <50 (<5 mg/dL) 4920 50,7% 889 36,2% 661 30,9% 0,000 0,000 0,000

2: 2. >50 (>5 mg/dL) 4779 49,3% 1570 63,8% 1476 69,1% 0,000 0,000 0,000

TOTAL 9699 2459 2137 0,000 0,000 0,000

----------------------------------------------------------------------------------------

Procalcitonina (0.5 ng/mL) p(ji2)= 0,000

A B C p(AB) p(AC) p(BC)

1: 1. <0.5 4145 89,6% 1105 84,2% 957 86,1% 0,000 0,001 0,209

2: 2. >0.5 480 10,4% 207 15,8% 155 13,9% 0,000 0,001 0,209

TOTAL 4625 1312 1112 0,000 0,001 0,206

----------------------------------------------------------------------------------------

LDH (300 U/L) p(ji2)= 0,000

A B C p(AB) p(AC) p(BC)

1: 1. <300 4184 48,3% 833 37,7% 609 30,1% 0,000 0,000 0,000

2: 2. >300 4470 51,7% 1375 62,3% 1414 69,9% 0,000 0,000 0,000

TOTAL 8654 2208 2023 0,000 0,000 0,000

----------------------------------------------------------------------------------------

----------------------------------------------------------------------------------------

Corticosteroides

A:No CS

B:Low-dose CS

C:CS megadoses

----------------------------------------------------------------------------------------

IL6 (4.3 pg/mL) p(ji2)= 0,228

A B C p(AB) p(AC) p(BC)

1: 1. <4.3 146 12,7% 37 10,4% 47 10,1% 0,266 0,150 0,908

2: 2. >4.3 1000 87,3% 320 89,6% 418 89,9% 0,266 0,150 0,908

TOTAL 1146 357 465 0,231 0,140 0,904

----------------------------------------------------------------------------------------

Dímero D (250 ng/mL) p(ji2)= 0,000

A B C p(AB) p(AC) p(BC)

1: 1. <250 1133 14,8% 182 8,9% 142 7,2% 0,000 0,000 0,049

2: 2. >250 6497 85,2% 1852 91,1% 1821 92,8% 0,000 0,000 0,049

TOTAL 7630 2034 1963 0,000 0,000 0,047

----------------------------------------------------------------------------------------

Fisher's chi2 overall and exact test for subgroups.

Unadjusted for multiple comparisons.

### Numerical variables

Corticosteroids

----------------------------------------------------------------------------------------------------

Nº Mean Std. Dev. P25 P50 P75 Swilk Levene t-test MW/KW

----------------------------------------------------------------------------------------------------

Hemoglobina (g/dL)

1:No CS 10042 13,72 1,856 12,7 13,9 15 0,000 0,000 0,000 0,000

2:Low-dose CS 2566 13,5 2 12,3 13,7 14,9 0,000

3:CS megadoses 2209 13,83 1,917 12,7 14 15,1 0,000

Plaquetas (x 10^6/L)

1:No CS 10047 207428 92308 149000 191000 247000 0,000 0,017 0,003 0,001

2:Low-dose CS 2563 201961 90659 144000 186000 241000 0,000

3:CS megadoses 2203 210757 96110 149000 189000 252000 0,000

Recuento de leucocitos (x10^6/L de sangre) al ingreso

1:No CS 10040 7137 5283 4700 6110 8200 0,000 0,000 0,000 0,000

2:Low-dose CS 2566 7917 5807 4950 6800 9300 0,000

3:CS megadoses 2209 7837 6077 5000 6600 9090 0,000

Recuento absoluto de neutrófilos (x10^6/L de sangre) al ingreso

1:No CS 9987 5262 4627 3100 4340 6300 0,000 0,000 0,000 0,000

2:Low-dose CS 2558 6125 4501 3500 5100 7670 0,000

3:CS megadoses 2201 6030 4472 3600 5100 7400 0,000

Recuento absoluto de linfocitos (x10^6/L de sangre) al ingreso

1:No CS 10026 1182 1981 700 1000 1350 0,000 0,056 0,423 0,000

2:Low-dose CS 2561 1156 2695 600 860 1200 0,000

3:CS megadoses 2206 1117 2362 600 850 1190 0,000

Proteína C reactiva al ingreso (mg/L)

1:No CS 9699 76,65 83,64 15,6 49 109,7 0,000 0,000 0,000 0,000

2:Low-dose CS 2459 105 93,55 29,4 82,82 157 0,000

3:CS megadoses 2137 114,2 96,73 36,3 93 164,3 0,000

Procalcitonina (ng/mL)

1:No CS 4625 ,4341 2,353 ,05 ,09 ,19 0,000 0,001 0,020 0,000

2:Low-dose CS 1312 ,6354 2,361 ,07 ,14 ,32 0,000

3:CS megadoses 1112 ,4706 1,991 ,075 ,13 ,26 0,000

LDH (U/L)

1:No CS 8654 355,7 207,6 237 306 413 0,000 0,000 0,000 0,000

2:Low-dose CS 2208 390,1 214 259 344 460 0,000

3:CS megadoses 2023 419,9 284,4 284 369 491 0,000

Interleukina-6 (IL-6) (pg/mL)

1:No CS 1146 54,92 160,4 10,1 24,24 49,4 0,000 0,000 0,000 0,000

2:Low-dose CS 357 82,57 158,1 13 41,4 87,6 0,000

3:CS megadoses 465 91,65 218,9 17,4 45 87,1 0,000

Dímero D (ng/mL)

1:No CS 7631 1643 7960 350 617 1174 0,000 0,000 0,000 0,000

2:Low-dose CS 2034 2458 12445 443 762 1405 0,000

3:CS megadoses 1963 2417 11879 468 780 1445 0,000

----------------------------------------------------------------------------------------------------

Swilk: prueba de normalidad Shapiro-Wilk

Levene: prueba de homogeneidad de varianzas

t-test: prueba t de student o ANOVA según corresponda

MW/KW: prueba Mann-Whitney o Kruskal-Wallis

Corticosteroids

A:No CS

B:Low-dose CS

C:CS megadoses

--------------------------------------------------------------------------------------

(A vs. B) (A vs. C) (B vs. C)

Swilk t-test MW t-test MW t-test MW

--------------------------------------------------------------------------------------

Hemoglobina (g/dL) 0,000 0,000 0,000 0,018 0,007 0,000 0,000

Plaquetas (x 10^6/L) 0,000 0,007 0,001 0,128 0,597 0,001 0,003

Recuento de leucocitos (x 0,000 0,000 0,000 0,000 0,000 0,640 0,259

Recuento absoluto de neut 0,000 0,000 0,000 0,000 0,000 0,462 0,622

Recuento absoluto de linf 0,000 0,580 0,000 0,177 0,000 0,598 0,754

Proteína C reactiva al i 0,000 0,000 0,000 0,000 0,000 0,001 0,000

Procalcitonina (ng/mL) 0,000 0,006 0,000 0,633 0,000 0,066 0,230

LDH (U/L) 0,000 0,000 0,000 0,000 0,000 0,000 0,000

Interleukina-6 (IL-6) (pg 0,000 0,004 0,000 0,000 0,000 0,508 0,386

Dímero D (ng/mL) 0,000 0,000 0,000 0,001 0,000 0,914 0,267

--------------------------------------------------------------------------------------

Swilk: prueba de normalidad Shapiro-Wilk

t-test: prueba t de Student

MW: prueba Mann-Whitney

Since they are not normal distributions, the Mann-Whitney test (last column) would be used. The p is not adjusted for Bonferroni.

## Bivariate Exitus in income

### Categorical variables

Exitus ingreso 0. No 1. Sí

-----------------------------------------------------------------------------------------------

Nº %Col Nº %Col %Fila Total Ji-2 p-exact

-----------------------------------------------------------------------------------------------

Sexo 11851 3055 14906 0,0000 0,0000

0: 0. Varón 6619 55,9% 1912 62,6% 22,4% 8531 57,2%

1: 1. Mujer 5232 44,1% 1143 37,4% 17,9% 6375 42,8%

-----------------------------------------------------------------------------------------------

Raza 11656 3022 14678 0,0000 0,0000

0: 0. Caucásica 10334 88,7% 2920 96,6% 22,0% 13254 90,3% 0,0000 0,0000

1: 1. Negra 51 0,4% 3 0,1% 5,6% 54 0,4% 0,0062 0,0037

2: 2. Latina 1096 9,4% 86 2,8% 7,3% 1182 8,1% 0,0000 0,0000

3: 3. Asiática 59 0,5% 4 0,1% 6,3% 63 0,4% 0,0051 0,0028

4: 4. Otras 116 1,0% 9 0,3% 7,2% 125 0,9% 0,0002 0,0001

-----------------------------------------------------------------------------------------------

Hipertensión arterial 11847 3052 14899 0,0000 0,0000

0: 0. No 6424 54,2% 902 29,6% 12,3% 7326 49,2%

1: 1. Sí 5423 45,8% 2150 70,4% 28,4% 7573 50,8%

-----------------------------------------------------------------------------------------------

Diabetes Mellitus 11826 3050 14876 0,0000 0,0000

0: 0. No 9833 83,1% 2179 71,4% 18,1% 12012 80,7%

1: 1. Sí 1993 16,9% 871 28,6% 30,4% 2864 19,3%

-----------------------------------------------------------------------------------------------

Diabetes sin lesión de � 11837 3053 14890 0,0000 0,0000

0: 0. No 10309 87,1% 2484 81,4% 19,4% 12793 85,9%

1: 1. Sí 1528 12,9% 569 18,6% 27,1% 2097 14,1%

-----------------------------------------------------------------------------------------------

Diabetes con lesión en � 11836 3053 14889 0,0000 0,0000

0: 0. No 11356 95,9% 2742 89,8% 19,4% 14098 94,7%

1: 1. Sí 480 4,1% 311 10,2% 39,3% 791 5,3%

-----------------------------------------------------------------------------------------------

Dislipemia 11843 3047 14890 0,0000 0,0000

0: 0. No 7483 63,2% 1505 49,4% 16,7% 8988 60,4%

1: 1. Sí 4360 36,8% 1542 50,6% 26,1% 5902 39,6%

-----------------------------------------------------------------------------------------------

Obesidad (IMC mayor o igu 10887 2686 13573 0,0155 0,0163

0: 0. No 8634 79,3% 2073 77,2% 19,4% 10707 78,9%

1: 1. Sí 2253 20,7% 613 22,8% 21,4% 2866 21,1%

-----------------------------------------------------------------------------------------------

Historia de tabaquismo 11358 2869 14227 0,0000 0,0000

0: 0. Nunca ha fumado 8053 70,9% 1806 62,9% 18,3% 9859 69,3% 0,0000 0,0000

1: 1. Exfumador 2687 23,7% 926 32,3% 25,6% 3613 25,4% 0,0000 0,0000

2: 2. Fumador 618 5,4% 137 4,8% 18,1% 755 5,3% 0,1551 0,1621

-----------------------------------------------------------------------------------------------

Enolismo 11511 2918 14429 0,0408 0,0445

0: 0. No 10991 95,5% 2760 94,6% 20,1% 13751 95,3%

1: 1. Sí 520 4,5% 158 5,4% 23,3% 678 4,7%

-----------------------------------------------------------------------------------------------

Fibrilación auricular 11834 3047 14881 0,0000 0,0000

0: 0. No 10838 91,6% 2380 78,1% 18,0% 13218 88,8%

1: 1. Sí 996 8,4% 667 21,9% 40,1% 1663 11,2%

-----------------------------------------------------------------------------------------------

Insuficiencia cardiaca 11844 3049 14893 0,0000 0,0000

0: 0. No 11242 94,9% 2580 84,6% 18,7% 13822 92,8%

1: 1. Sí 602 5,1% 469 15,4% 43,8% 1071 7,2%

-----------------------------------------------------------------------------------------------

Infarto de miocardio 11847 3052 14899 0,0000 0,0000

0: 0. No 11301 95,4% 2720 89,1% 19,4% 14021 94,1%

1: 1. Sí 546 4,6% 332 10,9% 37,8% 878 5,9%

-----------------------------------------------------------------------------------------------

Angina de pecho 11844 3051 14895 0,0000 0,0000

0: 0. No 11517 97,2% 2853 93,5% 19,9% 14370 96,5%

1: 1. Sí 327 2,8% 198 6,5% 37,7% 525 3,5%

-----------------------------------------------------------------------------------------------

Cardiopatía isquémica 11837 3049 14886 0,0000 0,0000

0: 0. No 11086 93,7% 2612 85,7% 19,1% 13698 92,0%

1: 1. Sí 751 6,3% 437 14,3% 36,8% 1188 8,0%

-----------------------------------------------------------------------------------------------

EPOC 11843 3050 14893 0,0000 0,0000

0: 0. No 11198 94,6% 2674 87,7% 19,3% 13872 93,1%

1: 1. Sí 645 5,4% 376 12,3% 36,8% 1021 6,9%

-----------------------------------------------------------------------------------------------

Bronquitis crónica 11839 3052 14891 0,0000 0,0000

0: 0. No 11364 96,0% 2781 91,1% 19,7% 14145 95,0%

1: 1. Sí 475 4,0% 271 8,9% 36,3% 746 5,0%

-----------------------------------------------------------------------------------------------

Exitus ingreso 0. No 1. Sí

-----------------------------------------------------------------------------------------------

Nº %Col Nº %Col %Fila Total Ji-2 p-exact

-----------------------------------------------------------------------------------------------

Asma 11842 3046 14888 0,0000 0,0000

0: 0. No 10915 92,2% 2894 95,0% 21,0% 13809 92,8%

1: 1. Sí 927 7,8% 152 5,0% 14,1% 1079 7,2%

-----------------------------------------------------------------------------------------------

Síndrome de apnea-hipopn 11783 3042 14825 0,0001 0,0002

0: 0. No 11125 94,4% 2816 92,6% 20,2% 13941 94,0%

1: 1. Sí 658 5,6% 226 7,4% 25,6% 884 6,0%

-----------------------------------------------------------------------------------------------

Ictus/AIT 11831 3042 14873 0,0000 0,0000

0: 0. No 11174 94,4% 2618 86,1% 19,0% 13792 92,7%

1: 1. Sí 657 5,6% 424 13,9% 39,2% 1081 7,3%

-----------------------------------------------------------------------------------------------

Accidente isquémico tran 11836 3049 14885 0,0000 0,0000

0: 0. No 11415 96,4% 2779 91,1% 19,6% 14194 95,4%

1: 1. Sí 421 3,6% 270 8,9% 39,1% 691 4,6%

-----------------------------------------------------------------------------------------------

ACV isquémico o hemorrá 11849 3047 14896 0,0000 0,0000

0: 0. No 11597 97,9% 2874 94,3% 19,9% 14471 97,1%

1: 1. Sí 252 2,1% 173 5,7% 40,7% 425 2,9%

-----------------------------------------------------------------------------------------------

Hemiplejia o paraplejia 11848 3050 14898 0,0000 0,0000

0: 0. No 11698 98,7% 2955 96,9% 20,2% 14653 98,4%

1: 1. Sí 150 1,3% 95 3,1% 38,8% 245 1,6%

-----------------------------------------------------------------------------------------------

Demencia 11837 3053 14890 0,0000 0,0000

0: 0. No 11029 93,2% 2365 77,5% 17,7% 13394 90,0%

1: 1. Sí 808 6,8% 688 22,5% 46,0% 1496 10,0%

-----------------------------------------------------------------------------------------------

Enfermedad neurológica d 11847 3050 14897 0,0000 0,0000

0: 0. No 11080 93,5% 2461 80,7% 18,2% 13541 90,9%

1: 1. Sí 767 6,5% 589 19,3% 43,4% 1356 9,1%

-----------------------------------------------------------------------------------------------

Hepatopatía crónica 11816 3044 14860 0,0003 0,0004

0: 0. No 11411 96,6% 2897 95,2% 20,2% 14308 96,3%

1: 1. Sí 405 3,4% 147 4,8% 26,6% 552 3,7%

-----------------------------------------------------------------------------------------------

Hepatopatía crónica lev 11834 3047 14881 0,0041 0,0054

0: 0. No 11528 97,4% 2939 96,5% 20,3% 14467 97,2%

1: 1. Sí 306 2,6% 108 3,5% 26,1% 414 2,8%

-----------------------------------------------------------------------------------------------

Hepatopatía crónica mod 11835 3052 14887 0,0096 0,0136

0: 0. No 11730 99,1% 3009 98,6% 20,4% 14739 99,0%

1: 1. Sí 105 0,9% 43 1,4% 29,1% 148 1,0%

-----------------------------------------------------------------------------------------------

Insuficiencia renal crón 11839 3049 14888 0,0000 0,0000

0: 0. No 11322 95,6% 2662 87,3% 19,0% 13984 93,9%

1: 1. Sí 517 4,4% 387 12,7% 42,8% 904 6,1%

-----------------------------------------------------------------------------------------------

Paciente habitualmente en 11822 3043 14865 0,0000 0,0000

0: 0. No 11713 99,1% 2979 97,9% 20,3% 14692 98,8% 0,0000 0,0000

1: 1. Hemodiálisis 93 0,8% 59 1,9% 38,8% 152 1,0% 0,0000 0,0000

2: 2. Diálisis periton 16 0,1% 5 0,2% 23,8% 21 0,1% 0,7044 0,7858

-----------------------------------------------------------------------------------------------

Enfermedad vascular perif 11836 3049 14885 0,0000 0,0000

0: 0. No 11416 96,5% 2769 90,8% 19,5% 14185 95,3%

1: 1. Sí 420 3,5% 280 9,2% 40,0% 700 4,7%

-----------------------------------------------------------------------------------------------

Úlcera gastroduodenal 11832 3051 14883 0,0000 0,0000

0: 0. No 11559 97,7% 2937 96,3% 20,3% 14496 97,4%

1: 1. Sí 273 2,3% 114 3,7% 29,5% 387 2,6%

-----------------------------------------------------------------------------------------------

Cáncer 11829 3049 14878 0,0000 0,0000

0: 0. No 10978 92,8% 2659 87,2% 19,5% 13637 91,7%

1: 1. Sí 851 7,2% 390 12,8% 31,4% 1241 8,3%

-----------------------------------------------------------------------------------------------

Neoplasia sólida sin met 11845 3055 14900 0,0000 0,0000

0: 0. No 11212 94,7% 2756 90,2% 19,7% 13968 93,7%

1: 1. Sí 633 5,3% 299 9,8% 32,1% 932 6,3%

-----------------------------------------------------------------------------------------------

Neoplasia sólida con met 11838 3050 14888 0,0000 0,0000

0: 0. No 11612 98,1% 2953 96,8% 20,3% 14565 97,8%

1: 1. Sí 226 1,9% 97 3,2% 30,0% 323 2,2%

-----------------------------------------------------------------------------------------------

Exitus ingreso 0. No 1. Sí

-----------------------------------------------------------------------------------------------

Nº %Col Nº %Col %Fila Total Ji-2 p-exact

-----------------------------------------------------------------------------------------------

Leucemia 11849 3054 14903 0,0000 0,0000

0: 0. No 11743 99,1% 2981 97,6% 20,2% 14724 98,8%

1: 1. Sí 106 0,9% 73 2,4% 40,8% 179 1,2%

-----------------------------------------------------------------------------------------------

Linfoma 11838 3054 14892 0,0000 0,0000

0: 0. No 11712 98,9% 2968 97,2% 20,2% 14680 98,6%

1: 1. Sí 126 1,1% 86 2,8% 40,6% 212 1,4%

-----------------------------------------------------------------------------------------------

Enfermedad del tejido con 11835 3048 14883 0,0005 0,0008

0: 0. No 11581 97,9% 2950 96,8% 20,3% 14531 97,6%

1: 1. Sí 254 2,1% 98 3,2% 27,8% 352 2,4%

-----------------------------------------------------------------------------------------------

Paciente con enfermedad r 11817 3042 14859 0,0043 0,0056

0: 0. No 11576 98,0% 2954 97,1% 20,3% 14530 97,8%

1: 1. Sí 241 2,0% 88 2,9% 26,7% 329 2,2%

-----------------------------------------------------------------------------------------------

Trastorno de ansiedad 11832 3033 14865 0,8943 0,9091

0: 0. No 10916 92,3% 2796 92,2% 20,4% 13712 92,2%

1: 1. Sí 916 7,7% 237 7,8% 20,6% 1153 7,8%

-----------------------------------------------------------------------------------------------

Depresión 11832 3031 14863 0,0000 0,0000

0: 0. No 10695 90,4% 2632 86,8% 19,7% 13327 89,7%

1: 1. Sí 1137 9,6% 399 13,2% 26,0% 1536 10,3%

-----------------------------------------------------------------------------------------------

SIDA (con criterios defin 11814 3052 14866 0,6251 0,5900

0: 0. No 11778 99,7% 3041 99,6% 20,5% 14819 99,7%

1: 1. Sí 36 0,3% 11 0,4% 23,4% 47 0,3%

-----------------------------------------------------------------------------------------------

Infección VIH conocida p 11812 3049 14861 0,2254 0,2680

0: 0. No 11726 99,3% 3033 99,5% 20,6% 14759 99,3%

1: 1. Sí 86 0,7% 16 0,5% 15,7% 102 0,7%

-----------------------------------------------------------------------------------------------

Inmunodepresores tto habi 11832 3042 14874 0,0070 0,0084

0: 0. No 11435 96,6% 2909 95,6% 20,3% 14344 96,4%

1: 1. Sí 397 3,4% 133 4,4% 25,1% 530 3,6%

-----------------------------------------------------------------------------------------------

Rapamicina (Sirolimus) 11813 3044 14857 0,9352 1,0000

0: 0. No 11777 99,7% 3035 99,7% 20,5% 14812 99,7%

1: 1. Sí 36 0,3% 9 0,3% 20,0% 45 0,3%

-----------------------------------------------------------------------------------------------

Terapias biológicas de b 11839 3048 14887 0,0527 0,0608

0: 0. No 11694 98,8% 2997 98,3% 20,4% 14691 98,7%

1: 1. Sí 145 1,2% 51 1,7% 26,0% 196 1,3%

-----------------------------------------------------------------------------------------------

Tratamiento habitual con 11838 3047 14885 0,0000 0,0000

0: 0. No 11407 96,4% 2825 92,7% 19,8% 14232 95,6%

1: 1. Sí 431 3,6% 222 7,3% 34,0% 653 4,4%

-----------------------------------------------------------------------------------------------

Tratamiento habitual con 11808 3035 14843 0,0000 0,0000

0: 0. No 10789 91,4% 2663 87,7% 19,8% 13452 90,6%

1: 1. Sí 1019 8,6% 372 12,3% 26,7% 1391 9,4%

-----------------------------------------------------------------------------------------------

SatO2 11539 2973 14512 0,0000 0,0000

0: 0. <90% 1838 15,9% 1469 49,4% 44,4% 3307 22,8%

1: 1. >90% 9701 84,1% 1504 50,6% 13,4% 11205 77,2%

-----------------------------------------------------------------------------------------------

SatO2 posterior 10045 2271 12316 0,0000 0,0000

0: 0. <90% 461 4,6% 1181 52,0% 71,9% 1642 13,3%

1: 1. >90% 9584 95,4% 1090 48,0% 10,2% 10674 86,7%

-----------------------------------------------------------------------------------------------

Condensación 11713 3026 14739 0,0000 0,0000

0: 0. No 6178 52,7% 1358 44,9% 18,0% 7536 51,1% 0,0000 0,0000

1: 1. Unilateral 2084 17,8% 497 16,4% 19,3% 2581 17,5% 0,0776 0,0812

2: 2. Bilateral 3451 29,5% 1171 38,7% 25,3% 4622 31,4% 0,0000 0,0000

-----------------------------------------------------------------------------------------------

Infiltrado intersticial/v 11722 3024 14746 0,0000 0,0000

0: 0. No 4399 37,5% 1110 36,7% 20,1% 5509 37,4% 0,4051 0,4110

1: 1. Unilateral 1307 11,1% 219 7,2% 14,4% 1526 10,3% 0,0000 0,0000

2: 2. Bilateral 6016 51,3% 1695 56,1% 22,0% 7711 52,3% 0,0000 0,0000

-----------------------------------------------------------------------------------------------

Derrame pleural 11713 3025 14738 0,0000 0,0000

0: 0. No 11288 96,4% 2766 91,4% 19,7% 14054 95,4% 0,0000 0,0000

1: 1. Unilateral 297 2,5% 154 5,1% 34,1% 451 3,1% 0,0000 0,0000

2: 2. Bilateral 128 1,1% 105 3,5% 45,1% 233 1,6% 0,0000 0,0000

-----------------------------------------------------------------------------------------------

Exitus ingreso 0. No 1. Sí

-----------------------------------------------------------------------------------------------

Nº %Col Nº %Col %Fila Total Ji-2 p-exact

-----------------------------------------------------------------------------------------------

¿Realizada TC torácica? 11765 3028 14793 0,0000 0,0000

0: 0. No 11011 93,6% 2904 95,9% 20,9% 13915 94,1%

1: 1. Sí 754 6,4% 124 4,1% 14,1% 878 5,9%

-----------------------------------------------------------------------------------------------

Condensación 9250 1863 11113 0,0000 0,0000

0: 0. No 4546 49,1% 613 32,9% 11,9% 5159 46,4% 0,0000 0,0000

1: 1. Unilateral 1325 14,3% 234 12,6% 15,0% 1559 14,0% 0,0455 0,0483

2: 2. Bilateral 3379 36,5% 1016 54,5% 23,1% 4395 39,5% 0,0000 0,0000

-----------------------------------------------------------------------------------------------

Infiltrado intersticial/v 9247 1857 11104 0,0000 0,0000

0: 0. No 3419 37,0% 490 26,4% 12,5% 3909 35,2% 0,0000 0,0000

1: 1. Unilateral 751 8,1% 74 4,0% 9,0% 825 7,4% 0,0000 0,0000

2: 2. Bilateral 5077 54,9% 1293 69,6% 20,3% 6370 57,4% 0,0000 0,0000

-----------------------------------------------------------------------------------------------

Derrame pleural 9229 1863 11092 0,0000 0,0000

0: 0. No 8903 96,5% 1673 89,8% 15,8% 10576 95,3% 0,0000 0,0000

1: 1. Unilateral 226 2,4% 113 6,1% 33,3% 339 3,1% 0,0000 0,0000

2: 2. Bilateral 100 1,1% 77 4,1% 43,5% 177 1,6% 0,0000 0,0000

-----------------------------------------------------------------------------------------------

¿Empeoramiento radiológ 9273 1862 11135 0,0000 0,0000

0: 0. No 6221 67,1% 517 27,8% 7,7% 6738 60,5%

1: 1. Sí 3052 32,9% 1345 72,2% 30,6% 4397 39,5%

-----------------------------------------------------------------------------------------------

Condensación al ingreso 11713 3026 14739 0,0000 0,0000

0: 0. No 6178 52,7% 1358 44,9% 18,0% 7536 51,1%

1: 1. Sí 5535 47,3% 1668 55,1% 23,2% 7203 48,9%

-----------------------------------------------------------------------------------------------

Infiltrado intesticial al 11722 3024 14746 0,4051 0,4110

0: 0. No 4399 37,5% 1110 36,7% 20,1% 5509 37,4%

1: 1. Sí 7323 62,5% 1914 63,3% 20,7% 9237 62,6%

-----------------------------------------------------------------------------------------------

Derrame pleural al ingres 11713 3025 14738 0,0000 0,0000

0: 0. No 11288 96,4% 2766 91,4% 19,7% 14054 95,4%

1: 1. Sí 425 3,6% 259 8,6% 37,9% 684 4,6%

-----------------------------------------------------------------------------------------------

Condensación en la evolu 9250 1863 11113 0,0000 0,0000

0: 0. No 4546 49,1% 613 32,9% 11,9% 5159 46,4%

1: 1. Sí 4704 50,9% 1250 67,1% 21,0% 5954 53,6%

-----------------------------------------------------------------------------------------------

Infiltrado intesticial en 9247 1857 11104 0,0000 0,0000

0: 0. No 3419 37,0% 490 26,4% 12,5% 3909 35,2%

1: 1. Sí 5828 63,0% 1367 73,6% 19,0% 7195 64,8%

-----------------------------------------------------------------------------------------------

Derrame pleural en la evo 9229 1863 11092 0,0000 0,0000

0: 0. No 8903 96,5% 1673 89,8% 15,8% 10576 95,3%

1: 1. Sí 326 3,5% 190 10,2% 36,8% 516 4,7%

-----------------------------------------------------------------------------------------------

Hemoglobina (12 g/dL) 11787 3030 14817 0,0000 0,0000

1: 1. <12 1734 14,7% 860 28,4% 33,2% 2594 17,5%

2: 2. >12 10053 85,3% 2170 71,6% 17,8% 12223 82,5%

-----------------------------------------------------------------------------------------------

Hemoglobina (12 g/dL) 11285 2524 13809 0,0000 0,0000

1: 1. <12 3110 27,6% 1023 40,5% 24,8% 4133 29,9%

2: 2. >12 8175 72,4% 1501 59,5% 15,5% 9676 70,1%

-----------------------------------------------------------------------------------------------

Leucocitosis (10 x 10^6/L 11787 3028 14815 0,0000 0,0000

1: 1. <10.000 10346 87,8% 2185 72,2% 17,4% 12531 84,6%

2: 2. >10.000 1441 12,2% 843 27,8% 36,9% 2284 15,4%

-----------------------------------------------------------------------------------------------

Leucocitos (4 x 10^6/L) 11787 3028 14815 0,0000 0,0000

1: 1. <4.000 1705 14,5% 341 11,3% 16,7% 2046 13,8%

2: 2. >4.000 10082 85,5% 2687 88,7% 21,0% 12769 86,2%

-----------------------------------------------------------------------------------------------

Leucocitosis (10 x 10^6/L 11289 2520 13809 0,0000 0,0000

1: 1. <10.000 9724 86,1% 1422 56,4% 12,8% 11146 80,7%

2: 2. >10.000 1565 13,9% 1098 43,6% 41,2% 2663 19,3%

-----------------------------------------------------------------------------------------------

Leucocitos (4 x 10^6/L) 11289 2520 13809 0,0000 0,0000

1: 1. <4.000 1426 12,6% 184 7,3% 11,4% 1610 11,7%

2: 2. >4.000 9863 87,4% 2336 92,7% 19,1% 12199 88,3%

-----------------------------------------------------------------------------------------------

Linfocitos (1.3 x 10^6/L) 11772 3021 14793 0,0000 0,0000

1: 1. <1300 8732 74,2% 2527 83,6% 22,4% 11259 76,1%

2: 2. >1300 3040 25,8% 494 16,4% 14,0% 3534 23,9%

-----------------------------------------------------------------------------------------------

Exitus ingreso 0. No 1. Sí

-----------------------------------------------------------------------------------------------

Nº %Col Nº %Col %Fila Total Ji-2 p-exact

-----------------------------------------------------------------------------------------------

Linfocitos (1.3 x 10^6/L) 11274 2514 13788 0,0000 0,0000

1: 1. <1300 6337 56,2% 2236 88,9% 26,1% 8573 62,2%

2: 2. >1300 4937 43,8% 278 11,1% 5,3% 5215 37,8%

-----------------------------------------------------------------------------------------------

Plaquetas (180 x 10^6/L) 11786 3027 14813 0,0000 0,0000

1: 1. <180 5035 42,7% 1566 51,7% 23,7% 6601 44,6%

2: 2. >180 6751 57,3% 1461 48,3% 17,8% 8212 55,4%

-----------------------------------------------------------------------------------------------

Plaquetas (180 x 10^6/L) 11273 2521 13794 0,0000 0,0000

1: 1. <180 1685 14,9% 970 38,5% 36,5% 2655 19,2%

2: 2. >180 9588 85,1% 1551 61,5% 13,9% 11139 80,8%

-----------------------------------------------------------------------------------------------

PCR (50 mg/L) 11417 2878 14295 0,0000 0,0000

1: 1. <50 (<5 mg/dL) 5660 49,6% 810 28,1% 12,5% 6470 45,3%

2: 2. >50 (>5 mg/dL) 5757 50,4% 2068 71,9% 26,4% 7825 54,7%

-----------------------------------------------------------------------------------------------

PCR (50 mg/L) 11001 2396 13397 0,0000 0,0000

1: 1. <50 (<5 mg/dL) 8252 75,0% 689 28,8% 7,7% 8941 66,7%

2: 2. >50 (>5 mg/dL) 2749 25,0% 1707 71,2% 38,3% 4456 33,3%

-----------------------------------------------------------------------------------------------

Procalcitonina (0.5 ng/mL 5664 1385 7049 0,0000 0,0000

1: 1. <0.5 5218 92,1% 989 71,4% 15,9% 6207 88,1%

2: 2. >0.5 446 7,9% 396 28,6% 47,0% 842 11,9%

-----------------------------------------------------------------------------------------------

Procalcitonina (0.5 ng/mL 4307 1069 5376 0,0000 0,0000

1: 1. <0.5 4076 94,6% 625 58,5% 13,3% 4701 87,4%

2: 2. >0.5 231 5,4% 444 41,5% 65,8% 675 12,6%

-----------------------------------------------------------------------------------------------

Ferritina (1000 mcg/L) 5095 811 5906 0,0000 0,0000

1: 1. <1000 3584 70,3% 456 56,2% 11,3% 4040 68,4%

2: 2. >1000 1511 29,7% 355 43,8% 19,0% 1866 31,6%

-----------------------------------------------------------------------------------------------

Ferritina (274 mcg/L) 5095 811 5906 0,0000 0,0000

1: 1. <274 1298 25,5% 133 16,4% 9,3% 1431 24,2%

2: 2. >274 3797 74,5% 678 83,6% 15,2% 4475 75,8%

-----------------------------------------------------------------------------------------------

Dímero D (250 ng/mL) 9527 2100 11627 0,0000 0,0000

1: 1. <250 1339 14,1% 118 5,6% 8,1% 1457 12,5%

2: 2. >250 8188 85,9% 1982 94,4% 19,5% 10170 87,5%

-----------------------------------------------------------------------------------------------

Dímero D (250 ng/mL) 8929 1708 10637 0,0000 0,0000

1: 1. <250 1285 14,4% 43 2,5% 3,2% 1328 12,5%

2: 2. >250 7644 85,6% 1665 97,5% 17,9% 9309 87,5%

-----------------------------------------------------------------------------------------------

IL6 (4.3 pg/mL) 1726 242 1968 0,0001 0,0000

1: 1. <4.3 220 12,7% 10 4,1% 4,3% 230 11,7%

2: 2. >4.3 1506 87,3% 232 95,9% 13,3% 1738 88,3%

-----------------------------------------------------------------------------------------------

IL6 (4.3 pg/mL) 1917 352 2269 0,0000 0,0000

1: 1. <4.3 503 26,2% 19 5,4% 3,6% 522 23,0%

2: 2. >4.3 1414 73,8% 333 94,6% 19,1% 1747 77,0%

-----------------------------------------------------------------------------------------------

LDH (300 U/L) 10442 2443 12885 0,0000 0,0000

1: 1. <300 5001 47,9% 625 25,6% 11,1% 5626 43,7%

2: 2. >300 5441 52,1% 1818 74,4% 25,0% 7259 56,3%

-----------------------------------------------------------------------------------------------

LDH (300 U/L) 10223 2116 12339 0,0000 0,0000

1: 1. <300 6417 62,8% 287 13,6% 4,3% 6704 54,3%

2: 2. >300 3806 37,2% 1829 86,4% 32,5% 5635 45,7%

-----------------------------------------------------------------------------------------------

Creatinina sérica (0.9 m 11752 3026 14778 0,0000 0,0000

1: 1. <0.9 6642 56,5% 857 28,3% 11,4% 7499 50,7%

2: 2. >0.9 5110 43,5% 2169 71,7% 29,8% 7279 49,3%

-----------------------------------------------------------------------------------------------

Creatinina sérica (0.9 m 11245 2513 13758 0,0000 0,0000

1: 1. <0.9 7595 67,5% 867 34,5% 10,2% 8462 61,5%

2: 2. >0.9 3650 32,5% 1646 65,5% 31,1% 5296 38,5%

-----------------------------------------------------------------------------------------------

Lopinavir/Ritonavir (LPV/ 11848 3053 14901 0,0000 0,0000

0: 0. No 4299 36,3% 1454 47,6% 25,3% 5753 38,6%

1: 1. Sí 7549 63,7% 1599 52,4% 17,5% 9148 61,4%

-----------------------------------------------------------------------------------------------

Exitus ingreso 0. No 1. Sí

-----------------------------------------------------------------------------------------------

Nº %Col Nº %Col %Fila Total Ji-2 p-exact

-----------------------------------------------------------------------------------------------

Hidroxicloroquina 11854 3057 14911 0,0000 0,0000

0: 0. No 1367 11,5% 772 25,3% 36,1% 2139 14,3%

1: 1. Sí 10487 88,5% 2285 74,7% 17,9% 12772 85,7%

-----------------------------------------------------------------------------------------------

Cloroquina 11823 3044 14867 0,5979 0,6246

0: 0. No 11284 95,4% 2912 95,7% 20,5% 14196 95,5%

1: 1. Sí 539 4,6% 132 4,3% 19,7% 671 4,5%

-----------------------------------------------------------------------------------------------

Tocilizumab 11831 3051 14882 0,0002 0,0003

0: 0. No 10883 92,0% 2742 89,9% 20,1% 13625 91,6%

1: 1. Sí 948 8,0% 309 10,1% 24,6% 1257 8,4%

-----------------------------------------------------------------------------------------------

Anakinra 11765 3022 14787 0,0461 0,0497

0: 0. No 11701 99,5% 2996 99,1% 20,4% 14697 99,4%

1: 1. Sí 64 0,5% 26 0,9% 28,9% 90 0,6%

-----------------------------------------------------------------------------------------------

Remdesivir 11781 3029 14810 0,4856 0,4513

0: 0. No 11730 99,6% 3013 99,5% 20,4% 14743 99,5%

1: 1. Sí 51 0,4% 16 0,5% 23,9% 67 0,5%

-----------------------------------------------------------------------------------------------

Interferón Beta-1B (IFNb 11803 3041 14844 0,0000 0,0000

0: 0. No 10678 90,5% 2505 82,4% 19,0% 13183 88,8%

1: 1. Sí 1125 9,5% 536 17,6% 32,3% 1661 11,2%

-----------------------------------------------------------------------------------------------

Colchicina 11640 3014 14654 0,0250 0,0312

0: 0. No 11544 99,2% 2976 98,7% 20,5% 14520 99,1%

1: 1. Sí 96 0,8% 38 1,3% 28,4% 134 0,9%

-----------------------------------------------------------------------------------------------

Inmunoglobulina 11648 3018 14666 0,0199 0,0248

0: 0. No 11601 99,6% 2996 99,3% 20,5% 14597 99,5%

1: 1. Sí 47 0,4% 22 0,7% 31,9% 69 0,5%

-----------------------------------------------------------------------------------------------

Baricitinib 9383 2457 11840 0,0672 0,0708

0: 0. No 9303 99,1% 2445 99,5% 20,8% 11748 99,2%

1: 1. Sí 80 0,9% 12 0,5% 13,0% 92 0,8%

-----------------------------------------------------------------------------------------------

Beclometasona inhalada 11723 3014 14737 0,0410 0,0425

0: 0. No 11139 95,0% 2836 94,1% 20,3% 13975 94,8%

1: 1. Sí 584 5,0% 178 5,9% 23,4% 762 5,2%

-----------------------------------------------------------------------------------------------

Corticoides sistémicos 11862 3059 14921 0,0000 0,0000

0: 0. No 8099 68,3% 1560 51,0% 16,2% 9659 64,7%

1: 1. Sí 3763 31,7% 1499 49,0% 28,5% 5262 35,3%

-----------------------------------------------------------------------------------------------

Anticoagulación oral dur 11812 3040 14852 0,0000 0,0000

0: 0. No 11426 96,7% 2903 95,5% 20,3% 14329 96,5% 0,0010 0,0013

1: 1. Antivitamina K 170 1,4% 83 2,7% 32,8% 253 1,7% 0,0000 0,0000

2: 2. ACOD 216 1,8% 54 1,8% 20,0% 270 1,8% 0,8473 0,9393

-----------------------------------------------------------------------------------------------

Heparina de bajo peso mol 11805 3041 14846 0,0000 0,0000

0: 0. No 1971 16,7% 634 20,8% 24,3% 2605 17,5% 0,0000 0,0000

1: 1. Dosis profilácti 7915 67,0% 1704 56,0% 17,7% 9619 64,8% 0,0000 0,0000

2: 2. Dosis plenas anti 1139 9,6% 489 16,1% 30,0% 1628 11,0% 0,0000 0,0000

3: 3. Dosis intermedias 780 6,6% 214 7,0% 21,5% 994 6,7% 0,3978 0,3931

-----------------------------------------------------------------------------------------------

Neumonía bacteriana 11836 3041 14877 0,0000 0,0000

0: 0. No 10881 91,9% 2342 77,0% 17,7% 13223 88,9%

1: 1. Sí 955 8,1% 699 23,0% 42,3% 1654 11,1%

-----------------------------------------------------------------------------------------------

Síndrome de distress res 11832 3025 14857 0,0000 0,0000

0: No/Leve 10440 88,2% 706 23,3% 6,3% 11146 75,0%

1: Moderado/Severo 1392 11,8% 2319 76,7% 62,5% 3711 25,0%

-----------------------------------------------------------------------------------------------

Insuficiencia cardiaca 11842 3046 14888 0,0000 0,0000

0: 0. No 11427 96,5% 2599 85,3% 18,5% 14026 94,2%

1: 1. Sí 415 3,5% 447 14,7% 51,9% 862 5,8%

-----------------------------------------------------------------------------------------------

Arritmia cardiaca 11835 3043 14878 0,0000 0,0000

0: 0. No 11536 97,5% 2770 91,0% 19,4% 14306 96,2%

1: 1. Sí 299 2,5% 273 9,0% 47,7% 572 3,8%

-----------------------------------------------------------------------------------------------

Arritmia cardiaca 11835 3043 14878 0,0000 0,0000

0: 0. No 11536 97,5% 2770 91,0% 19,4% 14306 96,2% 0,0000 0,0000

1: 1. Auriculares 273 2,3% 238 7,8% 46,6% 511 3,4% 0,0000 0,0000

2: 2. Ventriculares 14 0,1% 26 0,9% 65,0% 40 0,3% 0,0000 0,0000

3: 3. Ambas 12 0,1% 9 0,3% 42,9% 21 0,1% 0,0109 0,0247

-----------------------------------------------------------------------------------------------

Exitus ingreso 0. No 1. Sí

-----------------------------------------------------------------------------------------------

Nº %Col Nº %Col %Fila Total Ji-2 p-exact

-----------------------------------------------------------------------------------------------

Infarto de miocardio 11840 3043 14883 0,0000 0,0000

0: 0. No 11788 99,6% 2972 97,7% 20,1% 14760 99,2%

1: 1. Sí 52 0,4% 71 2,3% 57,7% 123 0,8%

-----------------------------------------------------------------------------------------------

Miocarditis 11843 3043 14886 0,0000 0,0000

0: 0. No 11777 99,4% 2974 97,7% 20,2% 14751 99,1%

1: 1. Sí 66 0,6% 69 2,3% 51,1% 135 0,9%

-----------------------------------------------------------------------------------------------

Crisis comiciales 11844 3047 14891 0,0002 0,0005

0: 0. No 11783 99,5% 3013 98,9% 20,4% 14796 99,4%

1: 1. Sí 61 0,5% 34 1,1% 35,8% 95 0,6%

-----------------------------------------------------------------------------------------------

Ictus 11837 3045 14882 0,0000 0,0000

0: 0. No 11785 99,6% 2993 98,3% 20,3% 14778 99,3%

1: 1. Sí 52 0,4% 52 1,7% 50,0% 104 0,7%

-----------------------------------------------------------------------------------------------

Ictus 11837 3045 14882 0,0000 0,0000

0: 0. No 11785 99,6% 2993 98,3% 20,3% 14778 99,3% 0,0000 0,0000

1: 1. Isquémico 47 0,4% 46 1,5% 49,5% 93 0,6% 0,0000 0,0000

2: 2. Hemorrágico 5 0,0% 6 0,2% 54,5% 11 0,1% 0,0051 0,0130

-----------------------------------------------------------------------------------------------

Insuficiencia renal aguda 11838 3044 14882 0,0000 0,0000

0: 0. No 10852 91,7% 1948 64,0% 15,2% 12800 86,0%

1: 1. Sí 986 8,3% 1096 36,0% 52,6% 2082 14,0%

-----------------------------------------------------------------------------------------------

Enfermedad tromboembólic 11827 3035 14862 0,6781 0,7236

0: 0. No 11566 97,8% 2979 98,2% 20,5% 14545 97,9% 0,2186 0,2317

1: 1. TVP 65 0,5% 14 0,5% 17,7% 79 0,5% 0,5506 0,6745

2: 2. TEP 178 1,5% 38 1,3% 17,6% 216 1,5% 0,2989 0,3494

3: 3. TVP+TEP 18 0,2% 4 0,1% 18,2% 22 0,1% 0,7943 1,0000

-----------------------------------------------------------------------------------------------

Enfermedad arterial perif 11784 3027 14811 0,0000 0,0000

0: 0. No 11740 99,6% 2994 98,9% 20,3% 14734 99,5%

1: 1. Sí 44 0,4% 33 1,1% 42,9% 77 0,5%

-----------------------------------------------------------------------------------------------

Sepsis 11836 3044 14880 0,0000 0,0000

0: 0. No 11548 97,6% 2414 79,3% 17,3% 13962 93,8%

1: 1. Sí 288 2,4% 630 20,7% 68,6% 918 6,2%

-----------------------------------------------------------------------------------------------

Coagulación intravascula 11829 3035 14864 0,0000 0,0000

0: 0. No 11770 99,5% 2926 96,4% 19,9% 14696 98,9%

1: 1. Sí 59 0,5% 109 3,6% 64,9% 168 1,1%

-----------------------------------------------------------------------------------------------

Shock 11826 3037 14863 0,0000 0,0000

0: 0. No 11656 98,6% 2531 83,3% 17,8% 14187 95,5%

1: 1. Sí 170 1,4% 506 16,7% 74,9% 676 4,5%

-----------------------------------------------------------------------------------------------

Fallo multiorgánico 11834 3038 14872 0,0000 0,0000

0: 0. No 11758 99,4% 2207 72,6% 15,8% 13965 93,9%

1: 1. Sí 76 0,6% 831 27,4% 91,6% 907 6,1%

-----------------------------------------------------------------------------------------------

Gafas nasales de alto flu 11782 3029 14811 0,0000 0,0000

0: 0. No 11015 93,5% 2607 86,1% 19,1% 13622 92,0%

1: 1. Sí 767 6,5% 422 13,9% 35,5% 1189 8,0%

-----------------------------------------------------------------------------------------------

Ventilación mecánica no 11829 3043 14872 0,0000 0,0000

0: 0. No 11478 97,0% 2675 87,9% 18,9% 14153 95,2%

1: 1. Sí 351 3,0% 368 12,1% 51,2% 719 4,8%

-----------------------------------------------------------------------------------------------

Ventilación mecánica in 11832 3047 14879 0,0000 0,0000

0: 0. No 11297 95,5% 2607 85,6% 18,8% 13904 93,4%

1: 1. Sí 535 4,5% 440 14,4% 45,1% 975 6,6%

-----------------------------------------------------------------------------------------------

¿Posición prono? 11818 3042 14860 0,0000 0,0000

0: 0. No 10964 92,8% 2377 78,1% 17,8% 13341 89,8%

1: 1. Sí 854 7,2% 665 21,9% 43,8% 1519 10,2%

-----------------------------------------------------------------------------------------------

Ingreso UCI 11856 3056 14912 0,0000 0,0000

0: 0. No 11119 93,8% 2575 84,3% 18,8% 13694 91,8%

1: 1. Sí 737 6,2% 481 15,7% 39,5% 1218 8,2%

-----------------------------------------------------------------------------------------------

¿Válido? 11862 3059 14921 0,0000 0,0000

0: 0. No 1337 11,3% 881 28,8% 39,7% 2218 14,9% 0,0000 0,0000

1: 1. Sí, completo 2944 24,8% 485 15,9% 14,1% 3429 23,0% 0,0000 0,0000

2: 2. Sí, con algunas 7581 63,9% 1693 55,3% 18,3% 9274 62,2% 0,0000 0,0000

-----------------------------------------------------------------------------------------------

Exitus ingreso 0. No 1. Sí

-----------------------------------------------------------------------------------------------

Nº %Col Nº %Col %Fila Total Ji-2 p-exact

-----------------------------------------------------------------------------------------------

Días de síntomas hasta 3603 1420 5023 0,0000 0,0000

1: 1. <10 días 1783 49,5% 936 65,9% 34,4% 2719 54,1%

2: 2. >10 días 1820 50,5% 484 34,1% 21,0% 2304 45,9%

-----------------------------------------------------------------------------------------------

Dosis máxima diaria de c 3596 1444 5040 0,2389 0,2476

1: 1. <125 mg 1919 53,4% 797 55,2% 29,3% 2716 53,9%

2: 2. >125 mg 1677 46,6% 647 44,8% 27,8% 2324 46,1%

-----------------------------------------------------------------------------------------------

Días de tratamiento con 3632 1442 5074 0,0000 0,0000

1: 1. <5 días 1871 51,5% 1031 71,5% 35,5% 2902 57,2%

2: 2. >5 días 1761 48,5% 411 28,5% 18,9% 2172 42,8%

-----------------------------------------------------------------------------------------------

Dosis acumulada en el ing 3417 1352 4769 0,0000 0,0000

1: 1. <500 mg 1583 46,3% 797 58,9% 33,5% 2380 49,9%

2: 2. >500 mg 1834 53,7% 555 41,1% 23,2% 2389 50,1%

-----------------------------------------------------------------------------------------------

Días con megadosis (Puls 3412 1382 4794 0,0163 0,0161

0: 0. No han recibido p 1795 52,6% 783 56,7% 30,4% 2578 53,8% 0,0109 0,0115

1: 1. <3 días 1298 38,0% 496 35,9% 27,6% 1794 37,4% 0,1631 0,1666

2: 2. >3 días 319 9,3% 103 7,5% 24,4% 422 8,8% 0,0358 0,0373

-----------------------------------------------------------------------------------------------

Uso de megadosis (Pulsos 3412 1382 4794 0,0109 0,0115

0: 0. No han recibido p 1795 52,6% 783 56,7% 30,4% 2578 53,8%

1: 1. Sí han recibido 1617 47,4% 599 43,3% 27,0% 2216 46,2%

-----------------------------------------------------------------------------------------------

Enf tromboembólica venos 11827 3035 14862 0,2186 0,2317

0: 0. No 11566 97,8% 2979 98,2% 20,5% 14545 97,9%

1: 1. Sí 261 2,2% 56 1,8% 17,7% 317 2,1%

-----------------------------------------------------------------------------------------------

Pruebas ji2 de Pearson y exacta de Fisher

.

### Numerical variables

----------------------------------------------------------------------------------------------------

Nº Mean Std. Dev. P25 P50 P75 Swilk Levene t-test MW/KW

----------------------------------------------------------------------------------------------------

Días de estancia hospitalaria en el ingreso

0:0. No 11862 12,78 162,5 6 9 14 0,000 0,516 0,282 0,000

1:1. Sí 3059 9,622 11,2 3 7 12 0,000

Días de síntomas hasta ingreso

0:0. No 11757 5,576 159,7 4 7 10 0,000 0,679 0,762 0,000

1:1. Sí 3003 4,693 9,05 2 5 7 0,000

Edad

0:0. No 11862 64,19 15,85 53,36 65 75,98 0,000 0,000 0,000 0,000

1:1. Sí 3059 79,67 10,58 74,34 81,39 86,91 0,000

Peso (kg)

0:0. No 5854 78,38 16,17 68 77 87 0,000 0,356 0,743 0,957

1:1. Sí 1300 78,54 17,21 67,85 77 87 0,000

Índice de Charlson

0:0. No 11580 1,073 1,647 0 0 2 0,000 0,000 0,000 0,000

1:1. Sí 2976 2,24 2,17 1 2 3 0,000

Charlson corregido por edad

0:0. No 11580 3,084 2,484 1 3 4 0,000 0,013 0,000 0,000

1:1. Sí 2976 5,697 2,417 4 5 7 0,000

FEV1 (%)

0:0. No 314 59,16 17,86 48 60 70 0,012 0,832 0,439 0,390

1:1. Sí 176 60,46 18,05 50 60,5 72,5 0,241

Saturación de oxígeno pulsioximetría (%)

0:0. No 11539 93,99 4,466 92 95 97 0,000 0,000 0,000 0,000

1:1. Sí 2973 88,65 8,582 86 91 94 0,000

FiO2 real si se dispone de ella (%)

0:0. No 50 53,78 32,89 21 40 100 0,004 0,532 0,043 0,082

1:1. Sí 21 71,76 35,12 31 100 100 0,087

pH en sangre arterial al ingreso

0:0. No 5833 7,439 ,1671 7,42 7,45 7,48 0,000 0,000 0,000 0,000

1:1. Sí 1829 7,414 ,2449 7,39 7,43 7,47 0,000

PCO2 al ingreso (mmHg)

0:0. No 5893 35,25 7,853 31 34 38,1 0,000 0,000 0,000 0,018

1:1. Sí 1854 36,53 11,27 30 34,55 40,5 0,000

PO2 al ingreso (mmHg)

0:0. No 5663 70,57 21,35 58,6 67,2 79 0,000 0,050 0,000 0,000

1:1. Sí 1742 62,76 22,85 49,9 59 70,6 0,000

pO2/FiO2 al ingreso (mmHg) (PO2/FiO2(%) x 100)

0:0. No 5422 304,2 94,16 253,6 300 352,4 0,000 0,192 0,000 0,000

1:1. Sí 1678 230,8 92,52 171,4 238,1 285,7 0,000

FiO2 real si se dispone de ella (%)

0:0. No 96 42,64 31,34 21 21 60 0,000 0,094 0,714 0,694

1:1. Sí 57 44,65 35 21 21 96 0,002

Saturación de oxígeno pulsioximetría (%)

0:0. No 10045 95,53 2,876 94 96 97 0,000 0,000 0,000 0,000

1:1. Sí 2271 88,37 8,669 85 90 94 0,000

FiO2 real si se dispone de ella (%)

0:0. No 225 69,06 24,67 50 60 100 0,000 0,008 0,000 0,000

1:1. Sí 237 80,5 22,12 60 90 100 0,000

pH en sangre arterial

0:0. No 2231 7,399 ,4182 7,39 7,43 7,46 0,000 0,015 0,001 0,000

1:1. Sí 835 7,342 ,3913 7,31 7,39 7,44 0,000

PCO2 (mmHg)

0:0. No 2244 40,95 10,13 35 39,3 45 0,000 0,000 0,000 0,000

1:1. Sí 840 45,09 15,31 35 42,05 52 0,000

PO2 (mmHg)

0:0. No 2185 80,27 26,88 64 75 91,2 0,000 0,000 0,000 0,000

1:1. Sí 812 72,93 30,06 52 65 86,55 0,000

pO2/FiO2 (mmHg) (PO2/FiO2(%) x 100)

0:0. No 2065 277 126,6 184,4 278,1 357,1 0,000 0,000 0,000 0,000

1:1. Sí 748 134,3 93,65 72,17 103,6 171,3 0,000

FiO2 real si se dispone de ella (%)

0:0. No 219 68,49 25,24 50 60 100 0,000 0,005 0,000 0,000

1:1. Sí 203 79,99 21,81 60 90 100 0,000

Hemoglobina (g/dL)

0:0. No 11787 13,83 1,786 12,8 14 15 0,000 0,000 0,000 0,000

1:1. Sí 3030 13,17 2,184 11,8 13,3 14,6 0,000

Recuento de leucocitos (x10^6/L de sangre) al ingreso

0:0. No 11787 7013 5020 4700 6100 8100 0,000 0,000 0,000 0,000

1:1. Sí 3028 8791 6931 5200 7365 10500 0,000

----------------------------------------------------------------------------------------------------

Nº Mean Std. Dev. P25 P50 P75 Swilk Levene t-test MW/KW

----------------------------------------------------------------------------------------------------

Recuento absoluto de neutrófilos (x10^6/L de sangre) al ingreso

0:0. No 11732 5158 4095 3100 4390 6210 0,000 0,000 0,000 0,000

1:1. Sí 3014 6960 5969 3800 5780 8730 0,000

Recuento absoluto de linfocitos (x10^6/L de sangre) al ingreso

0:0. No 11772 1175 1773 700 1000 1330 0,000 0,000 0,434 0,000

1:1. Sí 3021 1140 3317 500 770 1100 0,000

Plaquetas (x 10^6/L)

0:0. No 11786 210282 92487 151000 193000 250000 0,000 0,735 0,000 0,000

1:1. Sí 3027 194110 92080 137000 177000 233000 0,000

Proteína C reactiva al ingreso (mg/L)

0:0. No 11417 77,05 81,47 16,1 51 112 0,000 0,000 0,000 0,000

1:1. Sí 2878 127,2 104,1 42,7 105,7 184,6 0,000

Procalcitonina (ng/mL)

0:0. No 5664 ,317 1,637 ,05 ,09 ,17 0,000 0,000 0,000 0,000

1:1. Sí 1385 1,133 3,935 ,12 ,23 ,62 0,000

Ferritina sérica (mcg/L)

0:0. No 5095 892,4 1039 268 567 1164 0,000 0,000 0,000 0,000

1:1. Sí 811 1235 1376 397 803 1597 0,000

Dímero D (ng/mL)

0:0. No 9528 1652 9769 354 611,5 1099 0,000 0,000 0,000 0,000

1:1. Sí 2100 3115 8838 573,5 1113 2180 0,000

Interleukina-6 (IL-6) (pg/mL)

0:0. No 1726 60,51 173,8 10,3 26,3 55,7 0,000 0,001 0,000 0,000

1:1. Sí 242 126,5 183,5 39,2 78,25 146 0,000

LDH (U/L)

0:0. No 10442 347,1 193,9 239 307 405 0,000 0,000 0,000 0,000

1:1. Sí 2443 476,8 299,8 298 408 561 0,000

Límite superior normalidad LDH en su centro (U/L)

0:0. No 10577 263,2 77,27 225 245 250 0,000 0,000 0,000 0,000

1:1. Sí 2530 272,9 87,24 225 246 250 0,000

Creatinina sérica al ingreso (mg/dL)

0:0. No 11752 1,005 ,7578 ,71 ,87 1,07 0,000 0,000 0,000 0,000

1:1. Sí 3026 1,483 1,113 ,88 1,17 1,67 0,000

Urea (mg/dL)

0:0. No 9514 41,65 29,72 26 34 47 0,000 0,000 0,000 0,000

1:1. Sí 2426 74,46 51,35 42 59 90 0,000

Hemoglobina (g/dL)

0:0. No 11285 12,96 1,693 11,9 13,1 14,1 0,000 0,000 0,000 0,000

1:1. Sí 2524 12,52 2,163 11,1 12,6 14 0,000

Recuento de leucocitos (x10^6/L de sangre)

0:0. No 11289 7153 5034 4800 6200 8290 0,000 0,000 0,000 0,000

1:1. Sí 2520 10550 7350 6470 9200 12805 0,000

Recuento absoluto de neutrófilos (x10^6/L de sangre)

0:0. No 11240 4839 3748 2780 3961 5995 0,000 0,000 0,000 0,000

1:1. Sí 2511 8851 6423 5200 7800 11270 0,000

Recuento absoluto de linfocitos (x10^6/L de sangre)

0:0. No 11274 1404 2025 850 1220 1680 0,000 0,000 0,000 0,000

1:1. Sí 2514 1054 4196 400 600 900 0,000

Plaquetas (x 10^6/L)

0:0. No 11273 310723 135538 215000 292000 387000 0,000 0,000 0,000 0,000

1:1. Sí 2521 224961 112901 148000 207000 282000 0,000

Proteína C reactiva (mg/L)

0:0. No 11001 40,7 59,99 5,4 17,8 50 0,000 0,000 0,000 0,000

1:1. Sí 2396 141,8 114 40,63 123 218 0,000

Procalcitonina (ng/mL)

0:0. No 4307 ,2391 1,659 ,04 ,07 ,13 0,000 0,000 0,000 0,000

1:1. Sí 1069 1,85 5,291 ,16 ,36 1,25 0,000

Dímero D (ng/mL)

0:0. No 8929 1624 6716 359 637 1190 0,000 0,000 0,000 0,000

1:1. Sí 1708 6180 14004 821 1590 4400 0,000

Interleukina-6 (IL-6) (pg/mL)

0:0. No 1917 68,87 337,8 4,2 12 37,2 0,000 0,000 0,000 0,000

1:1. Sí 352 343,6 993,1 27,6 85 242,4 0,000

LDH (U/L)

0:0. No 10223 304,8 162,3 211 266 354 0,000 0,000 0,000 0,000

1:1. Sí 2116 594,7 450,8 364 497 687 0,000

Creatinina sérica (mg/dL)

0:0. No 11245 ,914 ,7149 ,67 ,8 ,97 0,000 0,000 0,000 0,000

1:1. Sí 2513 1,604 1,436 ,8 1,13 1,82 0,000

Urea (mg/dL)

0:0. No 8812 48,44 42,52 28 37 52 0,000 0,000 0,000 0,000

1:1. Sí 1956 94,98 68,51 48 75 120 0,000

GOT-AST(U/L)

0:0. No 9579 43,51 61,36 22 31 49 0,000 0,000 0,000 0,000

1:1. Sí 2059 78,81 274,6 30 44 68 0,000

----------------------------------------------------------------------------------------------------

Nº Mean Std. Dev. P25 P50 P75 Swilk Levene t-test MW/KW

----------------------------------------------------------------------------------------------------

GPT-ALT (U/L)

0:0. No 10651 57,3 72,18 22 37 67 0,000 0,108 0,071 0,000

1:1. Sí 2288 53,6 142,1 19 30 51 0,000

Glucemia basal(mg/dL)

0:0. No 10797 112 48,08 85 96 121 0,000 0,000 0,000 0,000

1:1. Sí 2402 157,1 80,03 105 135 185 0,000

Días desde el inicio de los síntomas hasta el inicio de corticoides sistémicos

0:0. No 3603 12,01 9,281 7 11 14 0,000 0,012 0,000 0,000

1:1. Sí 1420 10,25 10,55 5 8 12 0,000

Dosis máxima diaria de corticoides sistémicos (en mg de prednisona equivalentes)

0:0. No 3596 172,7 153,6 75 125 250 0,000 0,031 0,270 0,693

1:1. Sí 1444 178,3 180,6 75 125 250 0,000

Días de tratamiento con corticoides sistémicos

0:0. No 3632 7,506 6,033 3 5 10 0,000 0,000 0,000 0,000

1:1. Sí 1442 4,929 4,666 2 3 6 0,000

Dosis acumulada de corticoides durante el ingreso (en mg de prednisona equivalen

0:0. No 3417 703,6 591,7 300 562 937 0,000 0,232 0,000 0,000

1:1. Sí 1352 602,3 643,6 200 425 800 0,000

Número de días con megadosis de corticoides (más de 150 mg equivalentes en predn

0:0. No 3412 1,451 1,923 0 0 3 0,000 0,000 0,000 0,000

1:1. Sí 1382 1,187 1,724 0 0 3 0,000

Duración VMNI(días)

0:0. No 320 5,372 4,612 2 4 7 0,000 0,005 0,000 0,000

1:1. Sí 345 4,003 4,064 2 3 5 0,000

Duración VMI (días)

0:0. No 520 14,69 10,43 7,5 12 19 0,000 0,449 0,037 0,004

1:1. Sí 427 13,27 10,49 5 11 18 0,000

Número de días de ingreso en UCI

0:0. No 733 16,43 12,99 7 13 22 0,000 0,036 0,000 0,000

1:1. Sí 475 13,7 11,25 5 11 19 0,000

----------------------------------------------------------------------------------------------------

Swilk: prueba de normalidad Shapiro-Wilk

Levene: prueba de homogeneidad de varianzas

t-test: prueba t de student o ANOVA según corresponda

MW/KW: prueba Mann-Whitney o Kruskal-Wallis

## Bivariate Death during admission or re-admission

### Categorical variables

Death during admission or reinstatement

0. No 1. Sí

-----------------------------------------------------------------------------------------------

Nº %Col Nº %Col %Fila Total Ji-2 p-exact

-----------------------------------------------------------------------------------------------

Sexo 11620 3124 14744 0,0000 0,0000

0: 0. Varón 6500 55,9% 1948 62,4% 23,1% 8448 57,3%

1: 1. Mujer 5120 44,1% 1176 37,6% 18,7% 6296 42,7%

-----------------------------------------------------------------------------------------------

Raza 11429 3089 14518 0,0000 0,0000

0: 0. Caucásica 10131 88,6% 2986 96,7% 22,8% 13117 90,3% 0,0000 0,0000

1: 1. Negra 49 0,4% 3 0,1% 5,8% 52 0,4% 0,0062 0,0035

2: 2. Latina 1075 9,4% 86 2,8% 7,4% 1161 8,0% 0,0000 0,0000

3: 3. Asiática 58 0,5% 5 0,2% 7,9% 63 0,4% 0,0095 0,0079

4: 4. Otras 116 1,0% 9 0,3% 7,2% 125 0,9% 0,0001 0,0000

-----------------------------------------------------------------------------------------------

Hipertensión arterial 11616 3121 14737 0,0000 0,0000

0: 0. No 6318 54,4% 921 29,5% 12,7% 7239 49,1%

1: 1. Sí 5298 45,6% 2200 70,5% 29,3% 7498 50,9%

-----------------------------------------------------------------------------------------------

Diabetes Mellitus 11596 3119 14715 0,0000 0,0000

0: 0. No 9647 83,2% 2230 71,5% 18,8% 11877 80,7%

1: 1. Sí 1949 16,8% 889 28,5% 31,3% 2838 19,3%

-----------------------------------------------------------------------------------------------

Diabetes sin lesión de � 11607 3122 14729 0,0000 0,0000

0: 0. No 10113 87,1% 2537 81,3% 20,1% 12650 85,9%

1: 1. Sí 1494 12,9% 585 18,7% 28,1% 2079 14,1%

-----------------------------------------------------------------------------------------------

Diabetes con lesión en � 11606 3122 14728 0,0000 0,0000

0: 0. No 11136 96,0% 2809 90,0% 20,1% 13945 94,7%

1: 1. Sí 470 4,0% 313 10,0% 40,0% 783 5,3%

-----------------------------------------------------------------------------------------------

Dislipemia 11615 3115 14730 0,0000 0,0000

0: 0. No 7350 63,3% 1539 49,4% 17,3% 8889 60,3%

1: 1. Sí 4265 36,7% 1576 50,6% 27,0% 5841 39,7%

-----------------------------------------------------------------------------------------------

Obesidad (IMC mayor o igu 10677 2748 13425 0,0451 0,0466

0: 0. No 8455 79,2% 2128 77,4% 20,1% 10583 78,8%

1: 1. Sí 2222 20,8% 620 22,6% 21,8% 2842 21,2%

-----------------------------------------------------------------------------------------------

Historia de tabaquismo 11141 2934 14075 0,0000 0,0000

0: 0. Nunca ha fumado 7907 71,0% 1845 62,9% 18,9% 9752 69,3% 0,0000 0,0000

1: 1. Exfumador 2632 23,6% 948 32,3% 26,5% 3580 25,4% 0,0000 0,0000

2: 2. Fumador 602 5,4% 141 4,8% 19,0% 743 5,3% 0,1977 0,2103

-----------------------------------------------------------------------------------------------

Enolismo 11285 2984 14269 0,0615 0,0650

0: 0. No 10772 95,5% 2824 94,6% 20,8% 13596 95,3%

1: 1. Sí 513 4,5% 160 5,4% 23,8% 673 4,7%

-----------------------------------------------------------------------------------------------

Fibrilación auricular 11605 3116 14721 0,0000 0,0000

0: 0. No 10638 91,7% 2435 78,1% 18,6% 13073 88,8%

1: 1. Sí 967 8,3% 681 21,9% 41,3% 1648 11,2%

-----------------------------------------------------------------------------------------------

Insuficiencia cardiaca 11614 3118 14732 0,0000 0,0000

0: 0. No 11033 95,0% 2637 84,6% 19,3% 13670 92,8%

1: 1. Sí 581 5,0% 481 15,4% 45,3% 1062 7,2%

-----------------------------------------------------------------------------------------------

Infarto de miocardio 11617 3121 14738 0,0000 0,0000

0: 0. No 11089 95,5% 2782 89,1% 20,1% 13871 94,1%

1: 1. Sí 528 4,5% 339 10,9% 39,1% 867 5,9%

-----------------------------------------------------------------------------------------------

Angina de pecho 11615 3120 14735 0,0000 0,0000

0: 0. No 11297 97,3% 2917 93,5% 20,5% 14214 96,5%

1: 1. Sí 318 2,7% 203 6,5% 39,0% 521 3,5%

-----------------------------------------------------------------------------------------------

Cardiopatía isquémica 11608 3118 14726 0,0000 0,0000

0: 0. No 10876 93,7% 2673 85,7% 19,7% 13549 92,0%

1: 1. Sí 732 6,3% 445 14,3% 37,8% 1177 8,0%

-----------------------------------------------------------------------------------------------

Fallecimiento durante el ingreso o reingreso

0. No 1. Sí

-----------------------------------------------------------------------------------------------

Nº %Col Nº %Col %Fila Total Ji-2 p-exact

-----------------------------------------------------------------------------------------------

EPOC 11613 3119 14732 0,0000 0,0000

0: 0. No 10986 94,6% 2735 87,7% 19,9% 13721 93,1%

1: 1. Sí 627 5,4% 384 12,3% 38,0% 1011 6,9%

-----------------------------------------------------------------------------------------------

Bronquitis crónica 11610 3120 14730 0,0000 0,0000

0: 0. No 11148 96,0% 2843 91,1% 20,3% 13991 95,0%

1: 1. Sí 462 4,0% 277 8,9% 37,5% 739 5,0%

-----------------------------------------------------------------------------------------------

Asma 11613 3115 14728 0,0000 0,0000

0: 0. No 10706 92,2% 2957 94,9% 21,6% 13663 92,8%

1: 1. Sí 907 7,8% 158 5,1% 14,8% 1065 7,2%

-----------------------------------------------------------------------------------------------

Síndrome de apnea-hipopn 11554 3110 14664 0,0001 0,0001

0: 0. No 10909 94,4% 2877 92,5% 20,9% 13786 94,0%

1: 1. Sí 645 5,6% 233 7,5% 26,5% 878 6,0%

-----------------------------------------------------------------------------------------------

Ictus/AIT 11601 3111 14712 0,0000 0,0000

0: 0. No 10963 94,5% 2677 86,0% 19,6% 13640 92,7%

1: 1. Sí 638 5,5% 434 14,0% 40,5% 1072 7,3%

-----------------------------------------------------------------------------------------------

Accidente isquémico tran 11606 3118 14724 0,0000 0,0000

0: 0. No 11195 96,5% 2843 91,2% 20,3% 14038 95,3%

1: 1. Sí 411 3,5% 275 8,8% 40,1% 686 4,7%

-----------------------------------------------------------------------------------------------

ACV isquémico o hemorrá 11619 3116 14735 0,0000 0,0000

0: 0. No 11376 97,9% 2938 94,3% 20,5% 14314 97,1%

1: 1. Sí 243 2,1% 178 5,7% 42,3% 421 2,9%

-----------------------------------------------------------------------------------------------

Hemiplejia o paraplejia 11619 3119 14738 0,0000 0,0000

0: 0. No 11475 98,8% 3021 96,9% 20,8% 14496 98,4%

1: 1. Sí 144 1,2% 98 3,1% 40,5% 242 1,6%

-----------------------------------------------------------------------------------------------

Demencia 11607 3122 14729 0,0000 0,0000

0: 0. No 10831 93,3% 2415 77,4% 18,2% 13246 89,9%

1: 1. Sí 776 6,7% 707 22,6% 47,7% 1483 10,1%

-----------------------------------------------------------------------------------------------

Enfermedad neurológica d 11616 3119 14735 0,0000 0,0000

0: 0. No 10876 93,6% 2513 80,6% 18,8% 13389 90,9%

1: 1. Sí 740 6,4% 606 19,4% 45,0% 1346 9,1%

-----------------------------------------------------------------------------------------------

Hepatopatía crónica 11586 3113 14699 0,0001 0,0002

0: 0. No 11191 96,6% 2961 95,1% 20,9% 14152 96,3%

1: 1. Sí 395 3,4% 152 4,9% 27,8% 547 3,7%

-----------------------------------------------------------------------------------------------

Hepatopatía crónica lev 11604 3116 14720 0,0020 0,0026

0: 0. No 11306 97,4% 3004 96,4% 21,0% 14310 97,2%

1: 1. Sí 298 2,6% 112 3,6% 27,3% 410 2,8%

-----------------------------------------------------------------------------------------------

Hepatopatía crónica mod 11605 3121 14726 0,0092 0,0112

0: 0. No 11502 99,1% 3077 98,6% 21,1% 14579 99,0%

1: 1. Sí 103 0,9% 44 1,4% 29,9% 147 1,0%

-----------------------------------------------------------------------------------------------

Insuficiencia renal crón 11609 3118 14727 0,0000 0,0000

0: 0. No 11108 95,7% 2723 87,3% 19,7% 13831 93,9%

1: 1. Sí 501 4,3% 395 12,7% 44,1% 896 6,1%

-----------------------------------------------------------------------------------------------

Paciente habitualmente en 11591 3112 14703 0,0000 0,0000

0: 0. No 11487 99,1% 3045 97,8% 21,0% 14532 98,8% 0,0000 0,0000

1: 1. Hemodiálisis 90 0,8% 62 2,0% 40,8% 152 1,0% 0,0000 0,0000

2: 2. Diálisis periton 14 0,1% 5 0,2% 26,3% 19 0,1% 0,5824 0,5757

-----------------------------------------------------------------------------------------------

Enfermedad vascular perif 11607 3118 14725 0,0000 0,0000

0: 0. No 11199 96,5% 2832 90,8% 20,2% 14031 95,3%

1: 1. Sí 408 3,5% 286 9,2% 41,2% 694 4,7%

-----------------------------------------------------------------------------------------------

Úlcera gastroduodenal 11602 3120 14722 0,0000 0,0000

0: 0. No 11336 97,7% 3000 96,2% 20,9% 14336 97,4%

1: 1. Sí 266 2,3% 120 3,8% 31,1% 386 2,6%

-----------------------------------------------------------------------------------------------

Cáncer 11600 3118 14718 0,0000 0,0000

0: 0. No 10775 92,9% 2713 87,0% 20,1% 13488 91,6%

1: 1. Sí 825 7,1% 405 13,0% 32,9% 1230 8,4%

-----------------------------------------------------------------------------------------------

Fallecimiento durante el ingreso o reingreso

0. No 1. Sí

-----------------------------------------------------------------------------------------------

Nº %Col Nº %Col %Fila Total Ji-2 p-exact

-----------------------------------------------------------------------------------------------

Neoplasia sólida sin met 11615 3124 14739 0,0000 0,0000

0: 0. No 10994 94,7% 2820 90,3% 20,4% 13814 93,7%

1: 1. Sí 621 5,3% 304 9,7% 32,9% 925 6,3%

-----------------------------------------------------------------------------------------------

Neoplasia sólida con met 11609 3119 14728 0,0000 0,0000

0: 0. No 11400 98,2% 3009 96,5% 20,9% 14409 97,8%

1: 1. Sí 209 1,8% 110 3,5% 34,5% 319 2,2%

-----------------------------------------------------------------------------------------------

Leucemia 11620 3123 14743 0,0000 0,0000

0: 0. No 11518 99,1% 3047 97,6% 20,9% 14565 98,8%

1: 1. Sí 102 0,9% 76 2,4% 42,7% 178 1,2%

-----------------------------------------------------------------------------------------------

Linfoma 11608 3123 14731 0,0000 0,0000

0: 0. No 11487 99,0% 3034 97,2% 20,9% 14521 98,6%

1: 1. Sí 121 1,0% 89 2,8% 42,4% 210 1,4%

-----------------------------------------------------------------------------------------------

Enfermedad del tejido con 11605 3117 14722 0,0010 0,0014

0: 0. No 11354 97,8% 3018 96,8% 21,0% 14372 97,6%

1: 1. Sí 251 2,2% 99 3,2% 28,3% 350 2,4%

-----------------------------------------------------------------------------------------------

Paciente con enfermedad r 11588 3111 14699 0,0101 0,0135

0: 0. No 11349 97,9% 3023 97,2% 21,0% 14372 97,8%

1: 1. Sí 239 2,1% 88 2,8% 26,9% 327 2,2%

-----------------------------------------------------------------------------------------------

Trastorno de ansiedad 11601 3102 14703 0,6204 0,6226

0: 0. No 10712 92,3% 2856 92,1% 21,0% 13568 92,3%

1: 1. Sí 889 7,7% 246 7,9% 21,7% 1135 7,7%

-----------------------------------------------------------------------------------------------

Depresión 11602 3100 14702 0,0000 0,0000

0: 0. No 10500 90,5% 2689 86,7% 20,4% 13189 89,7%

1: 1. Sí 1102 9,5% 411 13,3% 27,2% 1513 10,3%

-----------------------------------------------------------------------------------------------

SIDA (con criterios defin 11587 3121 14708 0,5962 0,5850

0: 0. No 11553 99,7% 3110 99,6% 21,2% 14663 99,7%

1: 1. Sí 34 0,3% 11 0,4% 24,4% 45 0,3%

-----------------------------------------------------------------------------------------------

Infección VIH conocida p 11584 3118 14702 0,1855 0,2216

0: 0. No 11499 99,3% 3102 99,5% 21,2% 14601 99,3%

1: 1. Sí 85 0,7% 16 0,5% 15,8% 101 0,7%

-----------------------------------------------------------------------------------------------

Inmunodepresores tto habi 11602 3111 14713 0,0047 0,0055

0: 0. No 11214 96,7% 2974 95,6% 21,0% 14188 96,4%

1: 1. Sí 388 3,3% 137 4,4% 26,1% 525 3,6%

-----------------------------------------------------------------------------------------------

Rapamicina (Sirolimus) 11585 3112 14697 0,9687 1,0000

0: 0. No 11551 99,7% 3103 99,7% 21,2% 14654 99,7%

1: 1. Sí 34 0,3% 9 0,3% 20,9% 43 0,3%

-----------------------------------------------------------------------------------------------

Terapias biológicas de b 11609 3116 14725 0,0715 0,0760

0: 0. No 11467 98,8% 3065 98,4% 21,1% 14532 98,7%

1: 1. Sí 142 1,2% 51 1,6% 26,4% 193 1,3%

-----------------------------------------------------------------------------------------------

Tratamiento habitual con 11608 3115 14723 0,0000 0,0000

0: 0. No 11194 96,4% 2886 92,6% 20,5% 14080 95,6%

1: 1. Sí 414 3,6% 229 7,4% 35,6% 643 4,4%

-----------------------------------------------------------------------------------------------

Tratamiento habitual con 11578 3104 14682 0,0000 0,0000

0: 0. No 10579 91,4% 2723 87,7% 20,5% 13302 90,6%

1: 1. Sí 999 8,6% 381 12,3% 27,6% 1380 9,4%

-----------------------------------------------------------------------------------------------

SatO2 11319 3041 14360 0,0000 0,0000

0: 0. <90% 1798 15,9% 1485 48,8% 45,2% 3283 22,9%

1: 1. >90% 9521 84,1% 1556 51,2% 14,0% 11077 77,1%

-----------------------------------------------------------------------------------------------

SatO2 posterior 9853 2334 12187 0,0000 0,0000

0: 0. <90% 445 4,5% 1190 51,0% 72,8% 1635 13,4%

1: 1. >90% 9408 95,5% 1144 49,0% 10,8% 10552 86,6%

-----------------------------------------------------------------------------------------------

Condensación 11487 3093 14580 0,0000 0,0000

0: 0. No 6060 52,8% 1401 45,3% 18,8% 7461 51,2% 0,0000 0,0000

1: 1. Unilateral 2038 17,7% 511 16,5% 20,0% 2549 17,5% 0,1126 0,1156

2: 2. Bilateral 3389 29,5% 1181 38,2% 25,8% 4570 31,3% 0,0000 0,0000

-----------------------------------------------------------------------------------------------

Fallecimiento durante el ingreso o reingreso

0. No 1. Sí

-----------------------------------------------------------------------------------------------

Nº %Col Nº %Col %Fila Total Ji-2 p-exact

-----------------------------------------------------------------------------------------------

Infiltrado intersticial/v 11495 3092 14587 0,0000 0,0000

0: 0. No 4289 37,3% 1144 37,0% 21,1% 5433 37,2% 0,7492 0,7534

1: 1. Unilateral 1276 11,1% 228 7,4% 15,2% 1504 10,3% 0,0000 0,0000

2: 2. Bilateral 5930 51,6% 1720 55,6% 22,5% 7650 52,4% 0,0001 0,0001

-----------------------------------------------------------------------------------------------

Derrame pleural 11487 3093 14580 0,0000 0,0000

0: 0. No 11073 96,4% 2827 91,4% 20,3% 13900 95,3% 0,0000 0,0000

1: 1. Unilateral 287 2,5% 160 5,2% 35,8% 447 3,1% 0,0000 0,0000

2: 2. Bilateral 127 1,1% 106 3,4% 45,5% 233 1,6% 0,0000 0,0000

-----------------------------------------------------------------------------------------------

¿Realizada TC torácica? 11536 3097 14633 0,0000 0,0000

0: 0. No 10804 93,7% 2969 95,9% 21,6% 13773 94,1%

1: 1. Sí 732 6,3% 128 4,1% 14,9% 860 5,9%

-----------------------------------------------------------------------------------------------

Condensación 9082 1912 10994 0,0000 0,0000

0: 0. No 4467 49,2% 641 33,5% 12,5% 5108 46,5% 0,0000 0,0000

1: 1. Unilateral 1293 14,2% 243 12,7% 15,8% 1536 14,0% 0,0799 0,0816

2: 2. Bilateral 3322 36,6% 1028 53,8% 23,6% 4350 39,6% 0,0000 0,0000

-----------------------------------------------------------------------------------------------

Infiltrado intersticial/v 9077 1906 10983 0,0000 0,0000

0: 0. No 3342 36,8% 512 26,9% 13,3% 3854 35,1% 0,0000 0,0000

1: 1. Unilateral 729 8,0% 80 4,2% 9,9% 809 7,4% 0,0000 0,0000

2: 2. Bilateral 5006 55,2% 1314 68,9% 20,8% 6320 57,5% 0,0000 0,0000

-----------------------------------------------------------------------------------------------

Derrame pleural 9064 1911 10975 0,0000 0,0000

0: 0. No 8748 96,5% 1715 89,7% 16,4% 10463 95,3% 0,0000 0,0000

1: 1. Unilateral 219 2,4% 116 6,1% 34,6% 335 3,1% 0,0000 0,0000

2: 2. Bilateral 97 1,1% 80 4,2% 45,2% 177 1,6% 0,0000 0,0000

-----------------------------------------------------------------------------------------------

¿Empeoramiento radiológ 9103 1911 11014 0,0000 0,0000

0: 0. No 6108 67,1% 548 28,7% 8,2% 6656 60,4%

1: 1. Sí 2995 32,9% 1363 71,3% 31,3% 4358 39,6%

-----------------------------------------------------------------------------------------------

Condensación al ingreso 11487 3093 14580 0,0000 0,0000

0: 0. No 6060 52,8% 1401 45,3% 18,8% 7461 51,2%

1: 1. Sí 5427 47,2% 1692 54,7% 23,8% 7119 48,8%

-----------------------------------------------------------------------------------------------

Infiltrado intesticial al 11495 3092 14587 0,7492 0,7534

0: 0. No 4289 37,3% 1144 37,0% 21,1% 5433 37,2%

1: 1. Sí 7206 62,7% 1948 63,0% 21,3% 9154 62,8%

-----------------------------------------------------------------------------------------------

Derrame pleural al ingres 11487 3093 14580 0,0000 0,0000

0: 0. No 11073 96,4% 2827 91,4% 20,3% 13900 95,3%

1: 1. Sí 414 3,6% 266 8,6% 39,1% 680 4,7%

-----------------------------------------------------------------------------------------------

Condensación en la evolu 9082 1912 10994 0,0000 0,0000

0: 0. No 4467 49,2% 641 33,5% 12,5% 5108 46,5%

1: 1. Sí 4615 50,8% 1271 66,5% 21,6% 5886 53,5%

-----------------------------------------------------------------------------------------------

Infiltrado intesticial en 9077 1906 10983 0,0000 0,0000

0: 0. No 3342 36,8% 512 26,9% 13,3% 3854 35,1%

1: 1. Sí 5735 63,2% 1394 73,1% 19,6% 7129 64,9%

-----------------------------------------------------------------------------------------------

Derrame pleural en la evo 9064 1911 10975 0,0000 0,0000

0: 0. No 8748 96,5% 1715 89,7% 16,4% 10463 95,3%

1: 1. Sí 316 3,5% 196 10,3% 38,3% 512 4,7%

-----------------------------------------------------------------------------------------------

Hemoglobina (12 g/dL) 11558 3099 14657 0,0000 0,0000

1: 1. <12 1676 14,5% 890 28,7% 34,7% 2566 17,5%

2: 2. >12 9882 85,5% 2209 71,3% 18,3% 12091 82,5%

-----------------------------------------------------------------------------------------------

Hemoglobina (12 g/dL) 11074 2592 13666 0,0000 0,0000

1: 1. <12 3027 27,3% 1055 40,7% 25,8% 4082 29,9%

2: 2. >12 8047 72,7% 1537 59,3% 16,0% 9584 70,1%

-----------------------------------------------------------------------------------------------

Leucocitosis (10 x 10^6/L 11558 3097 14655 0,0000 0,0000

1: 1. <10.000 10145 87,8% 2243 72,4% 18,1% 12388 84,5%

2: 2. >10.000 1413 12,2% 854 27,6% 37,7% 2267 15,5%

-----------------------------------------------------------------------------------------------

Leucocitos (4 x 10^6/L) 11558 3097 14655 0,0000 0,0000

1: 1. <4.000 1677 14,5% 347 11,2% 17,1% 2024 13,8%

2: 2. >4.000 9881 85,5% 2750 88,8% 21,8% 12631 86,2%

-----------------------------------------------------------------------------------------------

Fallecimiento durante el ingreso o reingreso

0. No 1. Sí

-----------------------------------------------------------------------------------------------

Nº %Col Nº %Col %Fila Total Ji-2 p-exact

-----------------------------------------------------------------------------------------------

Leucocitosis (10 x 10^6/L 11078 2588 13666 0,0000 0,0000

1: 1. <10.000 9564 86,3% 1469 56,8% 13,3% 11033 80,7%

2: 2. >10.000 1514 13,7% 1119 43,2% 42,5% 2633 19,3%

-----------------------------------------------------------------------------------------------

Leucocitos (4 x 10^6/L) 11078 2588 13666 0,0000 0,0000

1: 1. <4.000 1401 12,6% 195 7,5% 12,2% 1596 11,7%

2: 2. >4.000 9677 87,4% 2393 92,5% 19,8% 12070 88,3%

-----------------------------------------------------------------------------------------------

Linfocitos (1.3 x 10^6/L) 11544 3089 14633 0,0000 0,0000

1: 1. <1300 8566 74,2% 2576 83,4% 23,1% 11142 76,1%

2: 2. >1300 2978 25,8% 513 16,6% 14,7% 3491 23,9%

-----------------------------------------------------------------------------------------------

Linfocitos (1.3 x 10^6/L) 11064 2582 13646 0,0000 0,0000

1: 1. <1300 6204 56,1% 2288 88,6% 26,9% 8492 62,2%

2: 2. >1300 4860 43,9% 294 11,4% 5,7% 5154 37,8%

-----------------------------------------------------------------------------------------------

Plaquetas (180 x 10^6/L) 11558 3096 14654 0,0000 0,0000

1: 1. <180 4939 42,7% 1602 51,7% 24,5% 6541 44,6%

2: 2. >180 6619 57,3% 1494 48,3% 18,4% 8113 55,4%

-----------------------------------------------------------------------------------------------

Plaquetas (180 x 10^6/L) 11063 2589 13652 0,0000 0,0000

1: 1. <180 1640 14,8% 995 38,4% 37,8% 2635 19,3%

2: 2. >180 9423 85,2% 1594 61,6% 14,5% 11017 80,7%

-----------------------------------------------------------------------------------------------

PCR (50 mg/L) 11193 2945 14138 0,0000 0,0000

1: 1. <50 (<5 mg/dL) 5537 49,5% 852 28,9% 13,3% 6389 45,2%

2: 2. >50 (>5 mg/dL) 5656 50,5% 2093 71,1% 27,0% 7749 54,8%

-----------------------------------------------------------------------------------------------

PCR (50 mg/L) 10797 2462 13259 0,0000 0,0000

1: 1. <50 (<5 mg/dL) 8108 75,1% 730 29,7% 8,3% 8838 66,7%

2: 2. >50 (>5 mg/dL) 2689 24,9% 1732 70,3% 39,2% 4421 33,3%

-----------------------------------------------------------------------------------------------

Procalcitonina (0.5 ng/mL 5533 1424 6957 0,0000 0,0000

1: 1. <0.5 5099 92,2% 1022 71,8% 16,7% 6121 88,0%

2: 2. >0.5 434 7,8% 402 28,2% 48,1% 836 12,0%

-----------------------------------------------------------------------------------------------

Procalcitonina (0.5 ng/mL 4221 1097 5318 0,0000 0,0000

1: 1. <0.5 3997 94,7% 648 59,1% 14,0% 4645 87,3%

2: 2. >0.5 224 5,3% 449 40,9% 66,7% 673 12,7%

-----------------------------------------------------------------------------------------------

Ferritina (1000 mcg/L) 4994 840 5834 0,0000 0,0000

1: 1. <1000 3509 70,3% 475 56,5% 11,9% 3984 68,3%

2: 2. >1000 1485 29,7% 365 43,5% 19,7% 1850 31,7%

-----------------------------------------------------------------------------------------------

Ferritina (274 mcg/L) 4994 840 5834 0,0000 0,0000

1: 1. <274 1260 25,2% 144 17,1% 10,3% 1404 24,1%

2: 2. >274 3734 74,8% 696 82,9% 15,7% 4430 75,9%

-----------------------------------------------------------------------------------------------

Dímero D (250 ng/mL) 9353 2145 11498 0,0000 0,0000

1: 1. <250 1319 14,1% 120 5,6% 8,3% 1439 12,5%

2: 2. >250 8034 85,9% 2025 94,4% 20,1% 10059 87,5%

-----------------------------------------------------------------------------------------------

Dímero D (250 ng/mL) 8777 1757 10534 0,0000 0,0000

1: 1. <250 1274 14,5% 45 2,6% 3,4% 1319 12,5%

2: 2. >250 7503 85,5% 1712 97,4% 18,6% 9215 87,5%

-----------------------------------------------------------------------------------------------

IL6 (4.3 pg/mL) 1684 250 1934 0,0001 0,0000

1: 1. <4.3 216 12,8% 11 4,4% 4,8% 227 11,7%

2: 2. >4.3 1468 87,2% 239 95,6% 14,0% 1707 88,3%

-----------------------------------------------------------------------------------------------

IL6 (4.3 pg/mL) 1878 363 2241 0,0000 0,0000

1: 1. <4.3 491 26,1% 23 6,3% 4,5% 514 22,9%

2: 2. >4.3 1387 73,9% 340 93,7% 19,7% 1727 77,1%

-----------------------------------------------------------------------------------------------

LDH (300 U/L) 10242 2499 12741 0,0000 0,0000

1: 1. <300 4902 47,9% 655 26,2% 11,8% 5557 43,6%

2: 2. >300 5340 52,1% 1844 73,8% 25,7% 7184 56,4%

-----------------------------------------------------------------------------------------------

LDH (300 U/L) 10035 2171 12206 0,0000 0,0000

1: 1. <300 6306 62,8% 315 14,5% 4,8% 6621 54,2%

2: 2. >300 3729 37,2% 1856 85,5% 33,2% 5585 45,8%

-----------------------------------------------------------------------------------------------

Fallecimiento durante el ingreso o reingreso

0. No 1. Sí

-----------------------------------------------------------------------------------------------

Nº %Col Nº %Col %Fila Total Ji-2 p-exact

-----------------------------------------------------------------------------------------------

Creatinina sérica (0.9 m 11525 3095 14620 0,0000 0,0000

1: 1. <0.9 6533 56,7% 884 28,6% 11,9% 7417 50,7%

2: 2. >0.9 4992 43,3% 2211 71,4% 30,7% 7203 49,3%

-----------------------------------------------------------------------------------------------

Creatinina sérica (0.9 m 11032 2581 13613 0,0000 0,0000

1: 1. <0.9 7467 67,7% 902 34,9% 10,8% 8369 61,5%

2: 2. >0.9 3565 32,3% 1679 65,1% 32,0% 5244 38,5%

-----------------------------------------------------------------------------------------------

Lopinavir/Ritonavir (LPV/ 11617 3122 14739 0,0000 0,0000

0: 0. No 4174 35,9% 1502 48,1% 26,5% 5676 38,5%

1: 1. Sí 7443 64,1% 1620 51,9% 17,9% 9063 61,5%

-----------------------------------------------------------------------------------------------

Hidroxicloroquina 11624 3126 14750 0,0000 0,0000

0: 0. No 1329 11,4% 795 25,4% 37,4% 2124 14,4%

1: 1. Sí 10295 88,6% 2331 74,6% 18,5% 12626 85,6%

-----------------------------------------------------------------------------------------------

Cloroquina 11596 3113 14709 0,8217 0,8458

0: 0. No 11071 95,5% 2975 95,6% 21,2% 14046 95,5%

1: 1. Sí 525 4,5% 138 4,4% 20,8% 663 4,5%

-----------------------------------------------------------------------------------------------

Tocilizumab 11601 3120 14721 0,0003 0,0004

0: 0. No 10678 92,0% 2809 90,0% 20,8% 13487 91,6%

1: 1. Sí 923 8,0% 311 10,0% 25,2% 1234 8,4%

-----------------------------------------------------------------------------------------------

Anakinra 11538 3090 14628 0,0327 0,0372

0: 0. No 11476 99,5% 3063 99,1% 21,1% 14539 99,4%

1: 1. Sí 62 0,5% 27 0,9% 30,3% 89 0,6%

-----------------------------------------------------------------------------------------------

Remdesivir 11553 3097 14650 0,4485 0,4439

0: 0. No 11505 99,6% 3081 99,5% 21,1% 14586 99,6%

1: 1. Sí 48 0,4% 16 0,5% 25,0% 64 0,4%

-----------------------------------------------------------------------------------------------

Interferón Beta-1B (IFNb 11575 3110 14685 0,0000 0,0000

0: 0. No 10466 90,4% 2571 82,7% 19,7% 13037 88,8%

1: 1. Sí 1109 9,6% 539 17,3% 32,7% 1648 11,2%

-----------------------------------------------------------------------------------------------

Colchicina 11417 3082 14499 0,0144 0,0176

0: 0. No 11326 99,2% 3043 98,7% 21,2% 14369 99,1%

1: 1. Sí 91 0,8% 39 1,3% 30,0% 130 0,9%

-----------------------------------------------------------------------------------------------

Inmunoglobulina 11422 3086 14508 0,0308 0,0382

0: 0. No 11375 99,6% 3064 99,3% 21,2% 14439 99,5%

1: 1. Sí 47 0,4% 22 0,7% 31,9% 69 0,5%

-----------------------------------------------------------------------------------------------

Baricitinib 9224 2515 11739 0,0545 0,0546

0: 0. No 9145 99,1% 2503 99,5% 21,5% 11648 99,2%

1: 1. Sí 79 0,9% 12 0,5% 13,2% 91 0,8%

-----------------------------------------------------------------------------------------------

Beclometasona inhalada 11497 3080 14577 0,0480 0,0536

0: 0. No 10927 95,0% 2900 94,2% 21,0% 13827 94,9%

1: 1. Sí 570 5,0% 180 5,8% 24,0% 750 5,1%

-----------------------------------------------------------------------------------------------

Corticoides sistémicos 11631 3128 14759 0,0000 0,0000

0: 0. No 7950 68,4% 1598 51,1% 16,7% 9548 64,7%

1: 1. Sí 3681 31,6% 1530 48,9% 29,4% 5211 35,3%

-----------------------------------------------------------------------------------------------

Anticoagulación oral dur 11582 3108 14690 0,0000 0,0000

0: 0. No 11206 96,8% 2967 95,5% 20,9% 14173 96,5% 0,0005 0,0008

1: 1. Antivitamina K 164 1,4% 86 2,8% 34,4% 250 1,7% 0,0000 0,0000

2: 2. ACOD 212 1,8% 55 1,8% 20,6% 267 1,8% 0,8217 0,8799

-----------------------------------------------------------------------------------------------

Heparina de bajo peso mol 11576 3109 14685 0,0000 0,0000

0: 0. No 1930 16,7% 648 20,8% 25,1% 2578 17,6% 0,0000 0,0000

1: 1. Dosis profilácti 7767 67,1% 1741 56,0% 18,3% 9508 64,7% 0,0000 0,0000

2: 2. Dosis plenas anti 1112 9,6% 503 16,2% 31,1% 1615 11,0% 0,0000 0,0000

3: 3. Dosis intermedias 767 6,6% 217 7,0% 22,1% 984 6,7% 0,4834 0,4922

-----------------------------------------------------------------------------------------------

Neumonía bacteriana 11605 3110 14715 0,0000 0,0000

0: 0. No 10685 92,1% 2397 77,1% 18,3% 13082 88,9%

1: 1. Sí 920 7,9% 713 22,9% 43,7% 1633 11,1%

-----------------------------------------------------------------------------------------------

Fallecimiento durante el ingreso o reingreso

0. No 1. Sí

-----------------------------------------------------------------------------------------------

Nº %Col Nº %Col %Fila Total Ji-2 p-exact

-----------------------------------------------------------------------------------------------

Síndrome de distress res 11601 3094 14695 0,0000 0,0000

0: No/Leve 10240 88,3% 766 24,8% 7,0% 11006 74,9%

1: Moderado/Severo 1361 11,7% 2328 75,2% 63,1% 3689 25,1%

-----------------------------------------------------------------------------------------------

Insuficiencia cardiaca 11611 3115 14726 0,0000 0,0000

0: 0. No 11219 96,6% 2661 85,4% 19,2% 13880 94,3%

1: 1. Sí 392 3,4% 454 14,6% 53,7% 846 5,7%

-----------------------------------------------------------------------------------------------

Arritmia cardiaca 11605 3112 14717 0,0000 0,0000

0: 0. No 11317 97,5% 2835 91,1% 20,0% 14152 96,2%

1: 1. Sí 288 2,5% 277 8,9% 49,0% 565 3,8%

-----------------------------------------------------------------------------------------------

Arritmia cardiaca 11605 3112 14717 0,0000 0,0000

0: 0. No 11317 97,5% 2835 91,1% 20,0% 14152 96,2% 0,0000 0,0000

1: 1. Auriculares 264 2,3% 242 7,8% 47,8% 506 3,4% 0,0000 0,0000

2: 2. Ventriculares 12 0,1% 26 0,8% 68,4% 38 0,3% 0,0000 0,0000

3: 3. Ambas 12 0,1% 9 0,3% 42,9% 21 0,1% 0,0148 0,0273

-----------------------------------------------------------------------------------------------

Infarto de miocardio 11610 3112 14722 0,0000 0,0000

0: 0. No 11559 99,6% 3041 97,7% 20,8% 14600 99,2%

1: 1. Sí 51 0,4% 71 2,3% 58,2% 122 0,8%

-----------------------------------------------------------------------------------------------

Miocarditis 11612 3112 14724 0,0000 0,0000

0: 0. No 11549 99,5% 3041 97,7% 20,8% 14590 99,1%

1: 1. Sí 63 0,5% 71 2,3% 53,0% 134 0,9%

-----------------------------------------------------------------------------------------------

Crisis comiciales 11613 3116 14729 0,0000 0,0001

0: 0. No 11556 99,5% 3079 98,8% 21,0% 14635 99,4%

1: 1. Sí 57 0,5% 37 1,2% 39,4% 94 0,6%

-----------------------------------------------------------------------------------------------

Ictus 11606 3114 14720 0,0000 0,0000

0: 0. No 11557 99,6% 3060 98,3% 20,9% 14617 99,3%

1: 1. Sí 49 0,4% 54 1,7% 52,4% 103 0,7%

-----------------------------------------------------------------------------------------------

Ictus 11606 3114 14720 0,0000 0,0000

0: 0. No 11557 99,6% 3060 98,3% 20,9% 14617 99,3% 0,0000 0,0000

1: 1. Isquémico 45 0,4% 47 1,5% 51,1% 92 0,6% 0,0000 0,0000

2: 2. Hemorrágico 4 0,0% 7 0,2% 63,6% 11 0,1% 0,0006 0,0028

-----------------------------------------------------------------------------------------------

Insuficiencia renal aguda 11608 3113 14721 0,0000 0,0000

0: 0. No 10663 91,9% 1999 64,2% 15,8% 12662 86,0%

1: 1. Sí 945 8,1% 1114 35,8% 54,1% 2059 14,0%

-----------------------------------------------------------------------------------------------

Enfermedad tromboembólic 11597 3104 14701 0,8285 0,8851

0: 0. No 11346 97,8% 3045 98,1% 21,2% 14391 97,9% 0,3640 0,3988

1: 1. TVP 63 0,5% 14 0,5% 18,2% 77 0,5% 0,5273 0,6741

2: 2. TEP 170 1,5% 41 1,3% 19,4% 211 1,4% 0,5463 0,6103

3: 3. TVP+TEP 18 0,2% 4 0,1% 18,2% 22 0,1% 0,7359 1,0000

-----------------------------------------------------------------------------------------------

Enfermedad arterial perif 11555 3096 14651 0,0000 0,0000

0: 0. No 11514 99,6% 3062 98,9% 21,0% 14576 99,5%

1: 1. Sí 41 0,4% 34 1,1% 45,3% 75 0,5%

-----------------------------------------------------------------------------------------------

Sepsis 11607 3113 14720 0,0000 0,0000

0: 0. No 11333 97,6% 2477 79,6% 17,9% 13810 93,8%

1: 1. Sí 274 2,4% 636 20,4% 69,9% 910 6,2%

-----------------------------------------------------------------------------------------------

Coagulación intravascula 11598 3104 14702 0,0000 0,0000

0: 0. No 11541 99,5% 2993 96,4% 20,6% 14534 98,9%

1: 1. Sí 57 0,5% 111 3,6% 66,1% 168 1,1%

-----------------------------------------------------------------------------------------------

Shock 11595 3106 14701 0,0000 0,0000

0: 0. No 11431 98,6% 2598 83,6% 18,5% 14029 95,4%

1: 1. Sí 164 1,4% 508 16,4% 75,6% 672 4,6%

-----------------------------------------------------------------------------------------------

Fallo multiorgánico 11603 3107 14710 0,0000 0,0000

0: 0. No 11531 99,4% 2272 73,1% 16,5% 13803 93,8%

1: 1. Sí 72 0,6% 835 26,9% 92,1% 907 6,2%

-----------------------------------------------------------------------------------------------

Gafas nasales de alto flu 11555 3097 14652 0,0000 0,0000

0: 0. No 10804 93,5% 2672 86,3% 19,8% 13476 92,0%

1: 1. Sí 751 6,5% 425 13,7% 36,1% 1176 8,0%

-----------------------------------------------------------------------------------------------

Fallecimiento durante el ingreso o reingreso

0. No 1. Sí

-----------------------------------------------------------------------------------------------

Nº %Col Nº %Col %Fila Total Ji-2 p-exact

-----------------------------------------------------------------------------------------------

Ventilación mecánica no 11599 3112 14711 0,0000 0,0000

0: 0. No 11256 97,0% 2743 88,1% 19,6% 13999 95,2%

1: 1. Sí 343 3,0% 369 11,9% 51,8% 712 4,8%

-----------------------------------------------------------------------------------------------

Ventilación mecánica in 11602 3116 14718 0,0000 0,0000

0: 0. No 11079 95,5% 2676 85,9% 19,5% 13755 93,5%

1: 1. Sí 523 4,5% 440 14,1% 45,7% 963 6,5%

-----------------------------------------------------------------------------------------------

¿Posición prono? 11588 3111 14699 0,0000 0,0000

0: 0. No 10749 92,8% 2445 78,6% 18,5% 13194 89,8%

1: 1. Sí 839 7,2% 666 21,4% 44,3% 1505 10,2%

-----------------------------------------------------------------------------------------------

Ingreso UCI 11625 3125 14750 0,0000 0,0000

0: 0. No 10906 93,8% 2644 84,6% 19,5% 13550 91,9%

1: 1. Sí 719 6,2% 481 15,4% 40,1% 1200 8,1%

-----------------------------------------------------------------------------------------------

¿Válido? 11631 3128 14759 0,0000 0,0000

0: 0. No 1167 10,0% 889 28,4% 43,2% 2056 13,9% 0,0000 0,0000

1: 1. Sí, completo 2926 25,2% 503 16,1% 14,7% 3429 23,2% 0,0000 0,0000

2: 2. Sí, con algunas 7538 64,8% 1736 55,5% 18,7% 9274 62,8% 0,0000 0,0000

-----------------------------------------------------------------------------------------------

Días de síntomas hasta 3527 1449 4976 0,0000 0,0000

1: 1. <10 días 1734 49,2% 957 66,0% 35,6% 2691 54,1%

2: 2. >10 días 1793 50,8% 492 34,0% 21,5% 2285 45,9%

-----------------------------------------------------------------------------------------------

Dosis máxima diaria de c 3519 1475 4994 0,1541 0,1615

1: 1. <125 mg 1874 53,3% 818 55,5% 30,4% 2692 53,9%

2: 2. >125 mg 1645 46,7% 657 44,5% 28,5% 2302 46,1%

-----------------------------------------------------------------------------------------------

Días de tratamiento con 3552 1473 5025 0,0000 0,0000

1: 1. <5 días 1836 51,7% 1044 70,9% 36,3% 2880 57,3%

2: 2. >5 días 1716 48,3% 429 29,1% 20,0% 2145 42,7%

-----------------------------------------------------------------------------------------------

Dosis acumulada en el ing 3344 1380 4724 0,0000 0,0000

1: 1. <500 mg 1549 46,3% 811 58,8% 34,4% 2360 50,0%

2: 2. >500 mg 1795 53,7% 569 41,2% 24,1% 2364 50,0%

-----------------------------------------------------------------------------------------------

Días con megadosis (Puls 3339 1411 4750 0,0094 0,0094

0: 0. No han recibido p 1753 52,5% 803 56,9% 31,4% 2556 53,8% 0,0053 0,0056

1: 1. <3 días 1274 38,2% 503 35,6% 28,3% 1777 37,4% 0,1028 0,1079

2: 2. >3 días 312 9,3% 105 7,4% 25,2% 417 8,8% 0,0342 0,0378

-----------------------------------------------------------------------------------------------

Uso de megadosis (Pulsos 3339 1411 4750 0,0053 0,0056

0: 0. No han recibido p 1753 52,5% 803 56,9% 31,4% 2556 53,8%

1: 1. Sí han recibido 1586 47,5% 608 43,1% 27,7% 2194 46,2%

-----------------------------------------------------------------------------------------------

Enf tromboembólica venos 11597 3104 14701 0,3640 0,3988

0: 0. No 11346 97,8% 3045 98,1% 21,2% 14391 97,9%

1: 1. Sí 251 2,2% 59 1,9% 19,0% 310 2,1%

-----------------------------------------------------------------------------------------------

Pruebas ji2 de Pearson y exacta de Fisher

### Numerical variables

----------------------------------------------------------------------------------------------------

Nº Mean Std. Dev. P25 P50 P75 Swilk Levene t-test MW/KW

----------------------------------------------------------------------------------------------------

Días de estancia hospitalaria en el ingreso

0:0. No 11631 12,77 164,1 6 9 14 0,000 0,537 0,304 0,000

1:1. Sí 3128 9,749 11,28 3 7 12 0,000

Días de síntomas hasta ingreso

0:0. No 11532 5,537 161,2 4 7 10 0,000 0,686 0,765 0,000

1:1. Sí 3068 4,665 9,037 2 5 7 0,000

Edad

0:0. No 11631 64,07 15,82 53,25 64,9 75,83 0,000 0,000 0,000 0,000

1:1. Sí 3128 79,67 10,57 74,35 81,39 86,92 0,000

Peso (kg)

0:0. No 5751 78,47 16,2 68 77 87 0,000 0,393 0,831 0,582

1:1. Sí 1329 78,36 17,2 67,8 77 87 0,000

Índice de Charlson

0:0. No 11358 1,06 1,627 0 0 1 0,000 0,000 0,000 0,000

1:1. Sí 3044 2,262 2,186 1 2 3 0,000

Charlson corregido por edad

0:0. No 11358 3,06 2,466 1 3 4 0,000 0,048 0,000 0,000

1:1. Sí 3044 5,718 2,418 4 5 7 0,000

FEV1 (%)

0:0. No 308 59,14 17,92 48 60 70 0,013 0,837 0,469 0,427

1:1. Sí 177 60,37 18,04 50 60 72 0,274

Saturación de oxígeno pulsioximetría (%)

0:0. No 11319 93,99 4,463 92 95 97 0,000 0,000 0,000 0,000

1:1. Sí 3041 88,76 8,545 86 91 95 0,000

FiO2 real si se dispone de ella (%)

0:0. No 50 53,78 32,89 21 40 100 0,004 0,532 0,043 0,082

1:1. Sí 21 71,76 35,12 31 100 100 0,087

pH en sangre arterial al ingreso

0:0. No 5720 7,439 ,1685 7,42 7,45 7,48 0,000 0,000 0,000 0,000

1:1. Sí 1860 7,414 ,243 7,39 7,43 7,47 0,000

PCO2 al ingreso (mmHg)

0:0. No 5776 35,24 7,845 31 34 38 0,000 0,000 0,000 0,007

1:1. Sí 1886 36,58 11,26 30 34,8 40,8 0,000

PO2 al ingreso (mmHg)

0:0. No 5551 70,54 21,24 58,8 67,2 79 0,000 0,023 0,000 0,000

1:1. Sí 1772 62,86 22,88 50 59 71 0,000

pO2/FiO2 al ingreso (mmHg) (PO2/FiO2(%) x 100)

0:0. No 5317 303,8 93,55 253,6 300 352,4 0,000 0,088 0,000 0,000

1:1. Sí 1706 232 93,1 172,5 238,1 285,7 0,000

FiO2 real si se dispone de ella (%)

0:0. No 92 43,54 31,71 21 21 65 0,000 0,272 0,996 0,527

1:1. Sí 60 43,52 34,46 21 21 88 0,000

Saturación de oxígeno pulsioximetría (%)

0:0. No 9853 95,54 2,824 94 96 97 0,000 0,000 0,000 0,000

1:1. Sí 2334 88,51 8,655 85 90 94 0,000

FiO2 real si se dispone de ella (%)

0:0. No 220 69,08 24,53 50 60 100 0,000 0,018 0,000 0,000

1:1. Sí 239 80,33 22,4 60 90 100 0,000

pH en sangre arterial

0:0. No 2195 7,399 ,4215 7,39 7,43 7,46 0,000 0,019 0,001 0,000

1:1. Sí 846 7,344 ,389 7,31 7,39 7,45 0,000

PCO2 (mmHg)

0:0. No 2205 40,94 10,14 35 39,3 45 0,000 0,000 0,000 0,000

1:1. Sí 852 45,04 15,26 35 42 51,95 0,000

PO2 (mmHg)

0:0. No 2152 80,29 26,72 64 75 91,05 0,000 0,000 0,000 0,000

1:1. Sí 823 72,82 29,97 52 65 86,4 0,000

pO2/FiO2 (mmHg) (PO2/FiO2(%) x 100)

0:0. No 2034 276,7 126,5 183,3 278,4 357,1 0,000 0,000 0,000 0,000

1:1. Sí 759 136,4 94,96 72,5 105 173,8 0,000

FiO2 real si se dispone de ella (%)

0:0. No 217 68,7 25,15 50 60 100 0,000 0,010 0,000 0,000

1:1. Sí 204 79,7 22,14 60 90 100 0,000

Hemoglobina (g/dL)

0:0. No 11558 13,85 1,777 12,8 14 15 0,000 0,000 0,000 0,000

1:1. Sí 3099 13,15 2,183 11,8 13,3 14,6 0,000

Recuento de leucocitos (x10^6/L de sangre) al ingreso

0:0. No 11558 7011 5035 4700 6100 8090 0,000 0,000 0,000 0,000

1:1. Sí 3097 8752 6871 5200 7330 10470 0,000

----------------------------------------------------------------------------------------------------

Nº Mean Std. Dev. P25 P50 P75 Swilk Levene t-test MW/KW

----------------------------------------------------------------------------------------------------

Recuento absoluto de neutrófilos (x10^6/L de sangre) al ingreso

0:0. No 11505 5157 4114 3100 4380 6200 0,000 0,000 0,000 0,000

1:1. Sí 3082 6926 5920 3800 5730 8700 0,000

Recuento absoluto de linfocitos (x10^6/L de sangre) al ingreso

0:0. No 11544 1176 1789 700 1000 1330 0,000 0,000 0,397 0,000

1:1. Sí 3089 1138 3282 500 770 1100 0,000

Plaquetas (x 10^6/L)

0:0. No 11558 210229 92300 151000 193000 250000 0,000 0,794 0,000 0,000

1:1. Sí 3096 194118 92060 137000 177000 233000 0,000

Proteína C reactiva al ingreso (mg/L)

0:0. No 11193 77,16 81,39 16,3 51 112,3 0,000 0,000 0,000 0,000

1:1. Sí 2945 126,1 104,5 41 103 183,6 0,000

Procalcitonina (ng/mL)

0:0. No 5533 ,3152 1,644 ,05 ,09 ,17 0,000 0,000 0,000 0,000

1:1. Sí 1424 1,113 3,886 ,11 ,23 ,61 0,000

Ferritina sérica (mcg/L)

0:0. No 4994 893,1 1033 271,7 570 1165 0,000 0,000 0,000 0,000

1:1. Sí 840 1234 1402 390,5 790,5 1590 0,000

Dímero D (ng/mL)

0:0. No 9354 1652 9847 354 610 1090 0,000 0,000 0,000 0,000

1:1. Sí 2145 3120 8792 577 1126 2190 0,000

Interleukina-6 (IL-6) (pg/mL)

0:0. No 1684 60,34 175,1 10,11 26,25 55,3 0,000 0,001 0,000 0,000

1:1. Sí 250 123,3 181,4 36,7 74,42 144,8 0,000

LDH (U/L)

0:0. No 10242 347,1 194,5 239 307 405 0,000 0,000 0,000 0,000

1:1. Sí 2499 474,2 298,5 294 406 558 0,000

Límite superior normalidad LDH en su centro (U/L)

0:0. No 10379 263,2 77,15 225 245 250 0,000 0,000 0,000 0,000

1:1. Sí 2585 272,9 87,19 225 246 250 0,000

Creatinina sérica al ingreso (mg/dL)

0:0. No 11525 1,001 ,7474 ,71 ,86 1,07 0,000 0,000 0,000 0,000

1:1. Sí 3095 1,482 1,119 ,88 1,17 1,67 0,000

Urea (mg/dL)

0:0. No 9326 41,5 29,68 26 34 46 0,000 0,000 0,000 0,000

1:1. Sí 2482 74,23 51,11 41 59 90 0,000

Hemoglobina (g/dL)

0:0. No 11074 12,97 1,688 11,9 13,1 14,1 0,000 0,000 0,000 0,000

1:1. Sí 2592 12,51 2,161 11 12,6 14 0,000

Recuento de leucocitos (x10^6/L de sangre)

0:0. No 11078 7136 5013 4800 6200 8240 0,000 0,000 0,000 0,000

1:1. Sí 2588 10486 7293 6400 9190 12785 0,000

Recuento absoluto de neutrófilos (x10^6/L de sangre)

0:0. No 11031 4825 3755 2770 3940 5940 0,000 0,000 0,000 0,000

1:1. Sí 2579 8788 6379 5133 7760 11200 0,000

Recuento absoluto de linfocitos (x10^6/L de sangre)

0:0. No 11064 1407 2041 860 1230 1680 0,000 0,000 0,000 0,000

1:1. Sí 2582 1052 4142 400 600 920 0,000

Plaquetas (x 10^6/L)

0:0. No 11063 311232 135613 215000 293000 387000 0,000 0,000 0,000 0,000

1:1. Sí 2589 225231 113105 148000 208000 282000 0,000

Proteína C reactiva (mg/L)

0:0. No 10797 40,63 59,99 5,4 17,7 50 0,000 0,000 0,000 0,000

1:1. Sí 2462 139,6 113,8 38,1 120 215,6 0,000

Procalcitonina (ng/mL)

0:0. No 4221 ,2348 1,655 ,04 ,07 ,13 0,000 0,000 0,000 0,000

1:1. Sí 1097 1,815 5,23 ,16 ,36 1,22 0,000

Dímero D (ng/mL)

0:0. No 8777 1623 6763 356 634 1187 0,000 0,000 0,000 0,000

1:1. Sí 1757 6074 13835 808 1582 4352 0,000

Interleukina-6 (IL-6) (pg/mL)

0:0. No 1878 68,34 339,9 4,2 12 37 0,000 0,000 0,000 0,000

1:1. Sí 363 338,7 979,7 26,8 83,9 244,9 0,000

LDH (U/L)

0:0. No 10035 304,2 161,7 211 266 353 0,000 0,000 0,000 0,000

1:1. Sí 2171 589,5 447,8 360 492 684 0,000

Creatinina sérica (mg/dL)

0:0. No 11032 ,9123 ,7155 ,67 ,8 ,97 0,000 0,000 0,000 0,000

1:1. Sí 2581 1,594 1,426 ,8 1,13 1,8 0,000

Urea (mg/dL)

0:0. No 8641 48,29 42,44 28 37 52 0,000 0,000 0,000 0,000

1:1. Sí 2011 94,35 68,22 48 74 119 0,000

GOT-AST(U/L)

0:0. No 9394 43,54 61,77 22 31 49 0,000 0,000 0,000 0,000

1:1. Sí 2113 77,81 271,2 29 44 67 0,000

----------------------------------------------------------------------------------------------------

Nº Mean Std. Dev. P25 P50 P75 Swilk Levene t-test MW/KW

----------------------------------------------------------------------------------------------------

GPT-ALT (U/L)

0:0. No 10453 57,38 72,48 22 37 67 0,000 0,177 0,039 0,000

1:1. Sí 2348 53,19 140,5 19 30 51 0,000

Glucemia basal(mg/dL)

0:0. No 10596 111,9 48,1 85 96 120 0,000 0,000 0,000 0,000

1:1. Sí 2467 156,2 79,49 104 134 184 0,000

Días desde el inicio de los síntomas hasta el inicio de corticoides sistémicos

0:0. No 3527 12,02 9,309 7 11 14 0,000 0,020 0,000 0,000

1:1. Sí 1449 10,22 10,48 5 8 12 0,000

Dosis máxima diaria de corticoides sistémicos (en mg de prednisona equivalentes)

0:0. No 3519 173,4 154,2 75 125 250 0,000 0,088 0,486 0,416

1:1. Sí 1475 176,9 179,3 75 125 250 0,000

Días de tratamiento con corticoides sistémicos

0:0. No 3552 7,481 6,013 3 5 10 0,000 0,000 0,000 0,000

1:1. Sí 1473 5,008 4,735 2 3 6 0,000

Dosis acumulada de corticoides durante el ingreso (en mg de prednisona equivalen

0:0. No 3344 705,1 594,3 300 570 937 0,000 0,367 0,000 0,000

1:1. Sí 1380 601,2 638,9 200 436 796,5 0,000

Número de días con megadosis de corticoides (más de 150 mg equivalentes en predn

0:0. No 3339 1,451 1,918 0 0 3 0,000 0,000 0,000 0,000

1:1. Sí 1411 1,184 1,721 0 0 3 0,000

Duración VMNI(días)

0:0. No 314 5,36 4,621 2 4 7 0,000 0,007 0,000 0,000

1:1. Sí 346 4,02 4,071 2 3 5 0,000

Duración VMI (días)

0:0. No 510 14,75 10,47 8 12 19 0,000 0,491 0,032 0,004

1:1. Sí 427 13,27 10,49 5 11 18 0,000

Número de días de ingreso en UCI

0:0. No 715 16,5 13,06 7 13 22 0,000 0,029 0,000 0,000

1:1. Sí 475 13,7 11,25 5 11 19 0,000

----------------------------------------------------------------------------------------------------

Swilk: prueba de normalidad Shapiro-Wilk

Levene: prueba de homogeneidad de varianzas

t-test: prueba t de student o ANOVA según corresponda

MW/KW: prueba Mann-Whitney o Kruskal-Wallis

## Multivariate analysis

### Exitus

#### Logistic Regression (univariate)

----------------------------------------------------------------------------------------------------

OR se z pvalue low_l upper_l PsR2 Nº AUC H-L

----------------------------------------------------------------------------------------------------

Exitus

Edad 1,088 ,00212 43,13 0,000 1,084 1,092 0,178 14921 0,791 0,000

Sexo ,7563 ,03155 -6,70 0,000 ,6969 ,8207 0,003 14906 0,534 .

Hipertensión arteri 2,824 ,1235 23,73 0,000 2,592 3,076 0,040 14899 0,623 .

Dislipemia 1,758 ,07199 13,79 0,000 1,623 1,905 0,013 14890 0,569 .

Fibrilación auricul 3,05 ,1675 20,30 0,000 2,738 3,396 0,025 14881 0,567 .

Insuficiencia cardia 3,395 ,2218 18,70 0,000 2,987 3,859 0,021 14893 0,551 .

EPOC 2,441 ,1669 13,06 0,000 2,135 2,791 0,010 14893 0,534 .

Ictus/AIT 2,754 ,1817 15,36 0,000 2,42 3,135 0,014 14873 0,542 .

Demencia 3,971 ,2248 24,36 0,000 3,554 4,437 0,037 14890 0,579 .

Enfermedad neurológ 3,457 ,2045 20,97 0,000 3,079 3,882 0,027 14897 0,564 .

Lopinavir/Ritonavir ,6263 ,02566 -11,42 0,000 ,578 ,6786 0,009 14901 0,557 .

Hidroxicloroquina ,3858 ,01952 -18,82 0,000 ,3494 ,426 0,022 14911 0,569 .

Tocilizumab 1,294 ,08914 3,74 0,000 1,13 1,481 0,001 14882 0,511 .

Baricitinib ,5707 ,1772 -1,81 0,071 ,3106 1,049 0,000 11840 0,502 .

Gafas nasales de alt 2,325 ,1497 13,10 0,000 2,049 2,637 0,011 14811 0,537 .

Ventilación mecáni 4,499 ,3493 19,37 0,000 3,864 5,238 0,023 14872 0,546 .

Ventilación mecáni 3,564 ,2421 18,71 0,000 3,12 4,071 0,022 14879 0,550 .

¿Posición prono? 3,592 ,2028 22,65 0,000 3,216 4,012 0,032 14860 0,573 .

Ingreso UCI 2,818 ,1763 16,56 0,000 2,493 3,186 0,017 14912 0,548 .

Uso de megadosis (Pu ,8492 ,05453 -2,55 0,011 ,7488 ,9631 0,001 4794 0,520 .

----------------------------------------------------------------------------------------------------

Corticosteroid doses are in three categories. The No-CS category is taken as reference.

Logistic regression Number of obs = 14.921

Wald chi2(2) = 301,35

Prob > chi2 = 0,0000

Log pseudolikelihood = -7421,3907 Pseudo R2 = 0,0195

-------------------------------------------------------------------------------

| Robust

Exitus | Odds Ratio Std. Err. z P>|z| [95% Conf. Interval]

--------------+----------------------------------------------------------------

grupo |

No CS | 1 (base)

Low-dose CS | 2,197967 ,1109714 15,60 0,000 1,990882 2,426592

CS megadoses | 1,866554 ,1022818 11,39 0,000 1,676475 2,078183

|

_cons | ,1984615 ,0053056 -60,49 0,000 ,1883304 ,2091376

-------------------------------------------------------------------------------

#### Multivariate

All of the above variables in a multivariate model:

Logistic regression Number of obs = 11.589

Wald chi2(21) = 2061,53

Prob > chi2 = 0,0000

Log pseudolikelihood = -4206,9238 Pseudo R2 = 0,2839

-------------------------------------------------------------------------------

| Robust

Exitus | Odds Ratio Std. Err. z P>|z| [95% Conf. Interval]

--------------+----------------------------------------------------------------

edad | 1,091674 ,0033779 28,35 0,000 1,085073 1,098314

sexo | ,585137 ,0352298 -8,90 0,000 ,5200064 ,6584252

hta | 1,211132 ,0760686 3,05 0,002 1,070852 1,369789

dislip | 1,086251 ,0616611 1,46 0,145 ,971878 1,214084

fauri | 1,204386 ,0941417 2,38 0,017 1,033311 1,403785

icc | 1,474407 ,1395961 4,10 0,000 1,22469 1,775042

epoc | 1,289617 ,1209253 2,71 0,007 1,073111 1,549803

acva | 1,251209 ,1110364 2,53 0,012 1,051457 1,48891

demencia | 1,304377 ,1419453 2,44 0,015 1,053837 1,614481

neudeg | 1,334989 ,1502965 2,57 0,010 1,070649 1,664593

kalet | ,9951957 ,0617721 -0,08 0,938 ,8811994 1,123939

hcq | ,500273 ,036951 -9,38 0,000 ,4328485 ,5782002

tocil | ,6295656 ,0770998 -3,78 0,000 ,4952204 ,8003566

baricit | ,3442117 ,1294562 -2,84 0,005 ,1646999 ,7193795

gafalf | 1,733182 ,1770701 5,38 0,000 1,41867 2,11742

vmni | 4,013622 ,4786911 11,65 0,000 3,176995 5,070565

vmi | 5,326794 1,418098 6,28 0,000 3,161252 8,975789

prono | 3,331302 ,3457921 11,59 0,000 2,718056 4,082907

uci | ,6881832 ,1789764 -1,44 0,151 ,4133627 1,145716

|

grupo |

No CS | 1 (base)

Low-dose CS | 1,397994 ,100614 4,66 0,000 1,214071 1,60978

CS megadoses | 1,536785 ,1218836 5,42 0,000 1,315539 1,795241

|

_cons | ,0003714 ,0000921 -31,86 0,000 ,0002285 ,0006038

-------------------------------------------------------------------------------

There would be no difference between megadoses and non-megadoses.

-------------------------------------------------------------------

| df chi2 P>chi2

--------------------------------+----------------------------------

grupo |

(No CS vs Low-dose CS ) | 1 21,67 0,0000

(CS megadoses vs Low-dose CS ) | 1 1,08 0,2989

Joint | 2 40,12 0,0000

-------------------------------------------------------------------

### Died during admission

#### Logistic Regression (univariate)

----------------------------------------------------------------------------------------------------

OR se z pvalue low_l upper_l PsR2 Nº AUC H-L

----------------------------------------------------------------------------------------------------

fallec

Edad 1,089 ,00214 43,44 0,000 1,085 1,093 0,182 14759 0,793 0,000

Sexo ,7664 ,03172 -6,43 0,000 ,7067 ,8312 0,003 14744 0,532 .

Hipertensión arteri 2,849 ,1238 24,10 0,000 2,616 3,102 0,041 14737 0,624 .

Dislipemia 1,765 ,07179 13,96 0,000 1,63 1,911 0,013 14730 0,569 .

Fibrilación auricul 3,077 ,1687 20,49 0,000 2,763 3,426 0,026 14721 0,568 .

Insuficiencia cardia 3,464 ,2264 19,01 0,000 3,047 3,937 0,022 14732 0,552 .

EPOC 2,46 ,1679 13,19 0,000 2,152 2,812 0,011 14732 0,535 .

Ictus/AIT 2,786 ,1835 15,56 0,000 2,448 3,17 0,015 14712 0,542 .

Demencia 4,086 ,2315 24,85 0,000 3,657 4,566 0,038 14729 0,580 .

Enfermedad neurológ 3,544 ,2094 21,41 0,000 3,157 3,979 0,028 14735 0,565 .

Lopinavir/Ritonavir ,6049 ,02462 -12,35 0,000 ,5585 ,6551 0,010 14739 0,561 .

Hidroxicloroquina ,3785 ,01906 -19,29 0,000 ,3429 ,4178 0,023 14750 0,570 .

Tocilizumab 1,281 ,08827 3,59 0,000 1,119 1,466 0,001 14721 0,510 .

Baricitinib ,555 ,1724 -1,90 0,058 ,3019 1,02 0,000 11739 0,502 .

Gafas nasales de alt 2,288 ,1474 12,85 0,000 2,017 2,596 0,010 14652 0,536 .

Ventilación mecáni 4,415 ,3442 19,04 0,000 3,789 5,143 0,023 14711 0,545 .

Ventilación mecáni 3,483 ,2375 18,30 0,000 3,047 3,981 0,021 14718 0,548 .

¿Posición prono? 3,49 ,1973 22,11 0,000 3,124 3,899 0,030 14699 0,571 .

Ingreso UCI 2,759 ,1732 16,17 0,000 2,44 3,121 0,016 14750 0,546 .

Uso de megadosis (Pu ,8369 ,05353 -2,78 0,005 ,7383 ,9487 0,001 4750 0,522 .

----------------------------------------------------------------------------------------------------

Logistic regression Number of obs = 14.759

Wald chi2(2) = 306,15

Prob > chi2 = 0,0000

Log pseudolikelihood = -7473,1669 Pseudo R2 = 0,0197

-------------------------------------------------------------------------------

| Robust

fallec | Odds Ratio Std. Err. z P>|z| [95% Conf. Interval]

--------------+----------------------------------------------------------------

grupo |

No CS | 1 (base)

Low-dose CS | 2,212192 ,1110282 15,82 0,000 2,004941 2,440866

CS megadoses | 1,851354 ,1010382 11,29 0,000 1,663546 2,060364

|

_cons | ,2070671 ,0054904 -59,39 0,000 ,1965808 ,2181126

-------------------------------------------------------------------------------

#### Multivariate

Logistic regression Number of obs = 11.493

Wald chi2(21) = 2070,26

Prob > chi2 = 0,0000

Log pseudolikelihood = -4239,1315 Pseudo R2 = 0,2850

-------------------------------------------------------------------------------

| Robust

fallec | Odds Ratio Std. Err. z P>|z| [95% Conf. Interval]

--------------+----------------------------------------------------------------

edad | 1,091047 ,0033448 28,42 0,000 1,084511 1,097622

sexo | ,5927978 ,0355139 -8,73 0,000 ,527123 ,666655

hta | 1,21988 ,0761087 3,19 0,001 1,07947 1,378554

dislip | 1,084421 ,0612747 1,43 0,151 ,9707359 1,21142

fauri | 1,206574 ,0939922 2,41 0,016 1,035727 1,405603

icc | 1,473899 ,1402281 4,08 0,000 1,223161 1,776036

epoc | 1,284033 ,1207381 2,66 0,008 1,067917 1,543885

acva | 1,249291 ,1105377 2,52 0,012 1,050386 1,485861

demencia | 1,297994 ,1404258 2,41 0,016 1,049987 1,60458

neudeg | 1,359542 ,1519679 2,75 0,006 1,09206 1,69254

kalet | ,9538363 ,0586913 -0,77 0,442 ,8454693 1,076093

hcq | ,4925738 ,0363113 -9,61 0,000 ,4263074 ,5691408

tocil | ,6439586 ,0783375 -3,62 0,000 ,507352 ,8173471

baricit | ,3359619 ,1261539 -2,90 0,004 ,1609398 ,7013208

gafalf | 1,740078 ,1760946 5,47 0,000 1,427012 2,121825

vmni | 3,866707 ,4601476 11,36 0,000 3,06229 4,882434

vmi | 5,291851 1,409378 6,26 0,000 3,139838 8,918831

prono | 3,263749 ,3367107 11,47 0,000 2,66625 3,995144

uci | ,6806079 ,1766894 -1,48 0,138 ,4091858 1,13207

|

grupo |

No CS | 1 (base)

Low-dose CS | 1,412079 ,1009097 4,83 0,000 1,227526 1,624379

CS megadoses | 1,531899 ,1207133 5,41 0,000 1,31267 1,787741

|

_cons | ,0004222 ,0001036 -31,68 0,000 ,0002611 ,0006828

-------------------------------------------------------------------------------

-------------------------------------------------------------------

| df chi2 P>chi2

--------------------------------+----------------------------------

grupo |

(No CS vs Low-dose CS ) | 1 23,32 0,0000

(CS megadoses vs Low-dose CS ) | 1 0,81 0,3680

Joint | 2 41,27 0,0000

-------------------------------------------------------------------
